# Supplementary material for: Self-rated health and perceived environmental quality in Brunei Darussalam: a cross-sectional study
Source: BMJ Open. 2022 Aug 18;12(8):e060799. doi: 10.1136/bmjopen-2022-060799 (PMC9394210; doi:10.1136/bmjopen-2022-060799)
Supplement: Author's manuscript [file bmjopen-2022-060799.draft_revisions.pdf]

BMJ Open is committed to open peer review. As part of this commitment we make the peer review history of every article we publish publicly available.

When an article is published we post the peer reviewers' comments and the authors' responses online. We also post the versions of the paper that were used during peer review. These are the versions that the peer review comments apply to.

The versions of the paper that follow are the versions that were submitted during the peer review process. They are not the versions of record or the final published versions. They should not be cited or distributed as the published version of this manuscript.

BMJ Open is an open access journal and the full, final, typeset and author-corrected version of record of the manuscript is available on our site with no access controls, subscription charges or pay-per-view fees (http://bmjopen.bmj.com).

If you have any questions on BMJ Open's open peer review process please email info.bmjopen@bmj.com

BMJ Open

Self-rated Health and Perceived Environmental Quality in Brunei Darussalam:A cross-sectional study

Journal:	*BMJ Open*
Manuscript ID	bmjopen-2022-060799
Article Type:	Original research
Date Submitted by the Author:	11-Jan-2022
Complete List of Authors:	Arifin, Evi; Universiti Brunei Darussalam, Centre for Advanced Research (CARe)<br>Hoon, Chang-Yau; Universiti Brunei Darussalam, Centre for Advanced Research (CARe)<br>Slesman, Ly; Universiti Brunei Darussalam, Centre for Advanced Research (CARe)<br>Tan, Abby; Universiti Brunei Darussalam, Chancellery
Keywords:	EPIDEMIOLOGY, PUBLIC HEALTH, PRIMARY CARE

SCHOLARONE™
Manuscripts

I, the Submitting Author has the right to grant and does grant on behalf of all authors of the Work (as defined in the below author licence), an exclusive licence and/or a non-exclusive licence for contributions from authors who are: i) UK Crown employees; ii) where BMJ has agreed a CC-BY licence shall apply, and/or iii) in accordance with the terms applicable for US Federal Government officers or employees acting as part of their official duties; on a worldwide, perpetual, irrevocable, royalty-free basis to BMJ Publishing Group Ltd ("BMJ") its licensees and where the relevant Journal is co-owned by BMJ to the co-owners of the Journal, to publish the Work in this journal and any other BMJ products and to exploit all rights, as set out in our licence.

The Submitting Author accepts and understands that any supply made under these terms is made by BMJ to the Submitting Author unless you are acting as an employee on behalf of your employer or a postgraduate student of an affiliated institution which is paying any applicable article publishing charge ("APC") for Open Access articles. Where the Submitting Author wishes to make the Work available on an Open Access basis (and intends to pay the relevant APC), the terms of reuse of such Open Access shall be governed by a Creative Commons licence – details of these licences and which Creative Commons licence will apply to this Work are set out in our licence referred to above.

Other than as permitted in any relevant BMJ Author's Self Archiving Policies, I confirm this Work has not been accepted for publication elsewhere, is not being considered for publication elsewhere and does not duplicate material already published. I confirm all authors consent to publication of this Work and authorise the granting of this licence.

For peer review only

Self-rated Health and Perceived Environmental Quality in Brunei Darussalam: A cross-sectional study

Evi Nurvidya Arifin*
Centre for Advanced Research (CARe), Universiti Brunei Darussalam,
Jalan Tungku Link, Gadong BE1410, Brunei Darussalam
Email: evi.arifin@ubd.edu.bn

Chang-Yau Hoon
Centre for Advanced Research (CARe), Universiti Brunei Darussalam,
Jalan Tungku Link, Gadong BE1410, Brunei Darussalam
Email: changyau.hoon@ubd.edu.bn

Ly Slesman
Centre for Advanced Research (CARe), Universiti Brunei Darussalam,
Jalan Tungku Link, Gadong BE1410, Brunei Darussalam
Email: slesman.ly@ubd.edu.bn

Abby Tan
Chancellery, Universiti Brunei Darussalam,
Jalan Tungku Link, Gadong BE1410, Brunei Darussalam
Email: abby.tan@ubd.edu.bn

Corresponding author: Evi Nurvidya Arifin Centre for Advanced Research (CARe), Universiti Brunei Darussalam, Jalan Tungku Link, Gadong BE1410, Brunei Darussalam. Email address: evi.arifin@ubd.edu.bn. Phone: +673 246 0922/3 Ext 2741.

ABSTRACT

Objectives: This paper examines the relationship between individual's perception on environmental quality and self-rated health (SRH), accounting for dimensions of socio-economic-demographic and healthy lifestyle variables.

Design: A cross-sectional survey.

Setting: It was conducted in Belait district, Brunei Darussalam between October and November 2019.

Participants: A total of 673 respondents were selected out of 1000 respondents aged 18 years and older.

Outcome measures: SRH was dichotomized with 1 for good health and 0 otherwise. Perception on environmental quality consisted of natural environment (air quality, marine quality, water supply, noise and olfactory pollution), and social environment (crime). Chi-square and logistic regression model were used to assess the relationship between individual's perceived environmental quality and SRH.

Results: Most respondents perceived themselves in good SRH (72%). The adjusted

For peer review only - http://bmjopen.bmj.com/site/about/guidelines.xhtml
For peer review only

logistic regression shows that the perception on the quality of air (OR=2.20, 95% CI 1.15-4.22, *p-value*=0.018) and marine resources (OR=1.84, 95% CI 1.24-2.74, *p-value*=0.002) in their surrounding areas were significantly associated with good SRH. However, other environmental variables were insignificantly associated with SRH. Among control variables, healthy lifestyle and employment had positive associations with good SRH (OR=3.89, 95% CI 1.96-7.71, *p-value*=0.000 for exercising 3-5 times a week, and OR=1.72, 95% CI 1.09-2.71, *p-value*=0.021 for being employed). In addition, frequent physical exercise compensated the negative health impact of environmental pollution.

Conclusions: This study suggests that environmental quality has an important role on SRH. However, healthy lifestyle measured with frequency of physical exercise seems to compensate the adverse environmental effects on SRH.

KEYWORDS: Self-rated health; perceived environmental quality; socio-economic-demographic condition; healthy lifestyle; Brunei Darussalam.

Article Summary

Strengths and limitations of this study

* This study's strength is in providing new empirical evidence on how environmental quality affects health, especially in oil- and gas-rich Belait district of Brunei Darussalam, one of the Southeast Asian countries.
* Another strength is that it used a cross-sectional survey with a representative and relatively large random sample of a district.
* One limitation is that perceptions on olfactory and noise pollutants were grouped into one category, therefore further studies should collect information separately for these pollutants.
* This study provided limited indicators of social environment and future studies should collect more indicators of social environment.
* Further studies should use this study as the base for a longitudinal study on the issue of environment and health to investigate the dynamics of the relationship.

For peer review only

INTRODUCTION

Ensuring healthy life is one of the 17 goals of sustainable development. Health crisis has far-reaching consequences on individuals, families, communities and states. At the individual level, being unhealthy encompasses suffering, deprivation in self-confidence and capability to function, as well as losing productivity and various opportunities. To some extent, being unhealthy can lead to a premature end of life.

What happen to individuals may impact the whole family. Unhealthy or sick family members may lead to family caregiving crisis, and disrupt social economic activities of other members of the family. They may even fall into poverty.

The communicable diseases, for example, may spread to the community. It may even become a pandemic, resulting in a sudden large disruption on people's life. At the state level, health crisis can weaken the heath-care system and destabilize the economy through contraction in employment, increase in state's expenditure and reduction in investment. The current COVID-19 pandemic provided evidence of health crisis impact on both health and economy in many countries around the globe.

To be healthy has multi-dimensional factors[1] with complex roles at different levels (individual, families, community and state). Previous studies were mostly concerned with health-related individual and family factors such as medical, biological or hereditary conditions; psychosocial, demographic, social and economic conditions, and healthy lifestyle [2],[3],[4],[5],[6],[7]. State level factors associated with health status were only considered when the analyses were concerned with cross-countries differences. For example, level of democracy was found to have an association with health[8]. Yet, analyses within a country or region should also

For peer review only - http://bmjopen.bmj.com/site/about/guidelines.xhtml

consider the health-related community factors especially the importance of surrounding environmental condition. This environmental condition can take three forms: built or man-made, natural and social environment[9].

Because it is not easy to measure objective measures of environmental quality at the individual level, this paper utilized its proxy -- perceived environmental quality. This variable was then used to examine the association between environmental quality of the surrounding areas and health status. Similarly, health status was assessed subjectively, known as self-rated health (SRH), on population-based surveys. SRH has been widely used in many countries. SRH is a reliable health indicator used in population-based studies [10], [11] and provides a reliable and acceptable approximation of the overall health outcomes of the population[11],[12],[13]. It captures biological, mental, and social states of an individual health[14] and has been shown to be able to explain mortality and morbidity [14], [15], and causes of deaths [7], [16].

Assessing the association between these two subjective domains have been conducted mostly in Western countries [17],[18],[19],[20],[21],[22], and some Asian countries such as Sri Lanka [23], China [9], [24], Japan and South Korea [25]. However, little is known on this relationship among Southeast Asian countries where countries in this region prospered and developed significantly. Natural environmental issues in this region such as seasonal forest fires have also been a transboundary concern[26].

Environment is a broad concept and has therefore been measured differently from one study to another. For example, a study in England and Scotland[17] measured neighbourhood environmental condition in relation to man-made, and

For peer review only - http://bmjopen.bmj.com/site/about/guidelines.xhtml

social environment such as access to private transportation, physical quality of residential environment, political climate and political engagement. Similarly, another study in the United Kingdom[21] used these environmental condition, but the selected variables referred to access to amenities, neighbourhood quality, and neighbourhood disorder. Unlike these studies [17], [21], environmental condition among residents of Chelsea, Massachusetts, US was measured differently by asking about perception on natural environment regarding air quality, odour and noise, and social environment including feeling safe, neighbourhood crime, and social cohesion[19]. A study in Sri Lanka[23] examined environmental factors using ventilation problems, water shortage, garbage disposal problems, mosquito threat, stray dogs, and social environment (nuisance from neighbours and overcrowding). A study in China concerning with its rapid development examined people's perception toward air pollution, water pollution, freshwater resource shortage and green space shortage in relation to self-related health[9].

However, findings from the abovementioned studies were inconclusive. The study in England and Scotland found that most of their selected neighbourhood environmental factors did not associate with SRH[17]. Similarly, none of the environmental factors were associated with SRH[23]. In contrast, some studies found that people's poor living environment was likely to be associated with higher likelihood of reporting poor SRH[18],[25],[19],[20],[21],[22]. Urban residents in China who perceived air pollution and noise pollution were more likely to report not in good SRH[24]. Air pollution had health impacts on respiratory diseases in some Southeast Asian countries[27] and non-communicable diseases such as chronic

For peer review only - http://bmjopen.bmj.com/site/about/guidelines.xhtml

kidney diseases in 194 countries and territories[28]. Different results were also found in China[24], that perceived environmental pollution was not significantly associated with SRH among rural residents of China. Nevertheless, there has been an insufficient number of empirical studies which assessed impacts of perceived environmental quality on self-rated health among Southeast Asian countries.

With this background, identifying environmental factors on health is crucial to contribute new empirical findings to the debate on the relationship between perceived environmental quality and self-rated health (SRH). This study aims to fill in the gap on this relationship by controlling for demographic, socio-economic and lifestyle variables in Brunei Darussalam (hereafter, Brunei). This study does not consider built environmental quality[9] due to unavailability of the information. Our primary objectives are to estimate the prevalence of good SRH, and to examine the relationship between natural and social environmental qualities and SRH accounting for dimensions of socio-economic-demographic and healthy lifestyle variables based on a cross-sectional study.

Brunei has provided its people with high quality of life as indicated with high HDI (Human Development Index) which stood at the 43th worldwide in 2018[29]. Its economy is heavily dependent on oil and gas production, with oil and natural gas exports as its major export and its main source of development and wealth since the mid-20th century. Brunei benefits considerably from the world market oil price, making it the second highest per capita GDP after Singapore among Southeast Asian countries.

For peer review only - http://bmjopen.bmj.com/site/about/guidelines.xhtml

METHODS

Design, Setting, Study size and Participants

We used the Strengthening the Reporting of Observational Studies in Epidemiology (STROBE) cross sectional checklist when writing our report[30]. The paper used an original cross-sectional survey conducted by the Universiti Brunei Darussalam in October-November 2019, before the COVID-19 pandemic spread to this country in March 2020. The survey was conducted in Belait district, the largest one of the four districts in Brunei in terms of land size. This district was home to around 75,900 persons, or 16.5% of 459,500 Brunei's population in 2019[31]. Within this district, three sub-districts were selected namely Seria, Liang, and Kuala Belait where majority of population reside and the major sources of the country's economy – the production of hydrocarbon resources - are located. They are spreading along the northern coastal area facing the South China Sea as seen in Figure 1.

Figure 1 about here

This survey interviewed 1,000 randomly selected respondents aged 18 years and above. Face-to-face interviews were performed by 11 trained research assistants. The survey gathered comprehensive information using a pre-tested structured questionnaire, that covered background characteristics, employment and income, housing, education and aspirations, health, and environmental impact and awareness. The questions were mostly closed-ended, combined with several open-ended ones. Prior to interviews, respondents were aware and informed about the objectives of the survey. The response for each selected variable varied due to various reasons such as

For peer review only

do not want to answer, do not know, unclear reason and others. For consistency, 673 respondents who completely responded to all questions were selected for this paper.

Patient and Public Involvement

Informed consent was provided for which respondents signed a consent form to agree or not to participate voluntarily.

Research Ethics Approval

Its research ethic was approved by the University Research Ethics Committee at Universiti Brunei Darussalam (ref: UBD/OAVCR/UREC/Sep19-04).

Variables and Measurements

The Dependent Variable: Self-rated Health (SRH)

Self-rated health (SRH) has been widely used in population-based surveys due to its simplicity as a singular question. Usually, the question took a form such as "In general, would you say that your health is …? [32], [33]. The Likert scale was usually used for the response. However, in practice the question varies slightly in different contexts. For instance, "Would you say your overall physical health is…?" was used by a study in the US [13]. Studies in Japan and Korea used "How would you rate your health?"[10], and in Norway "How is your health at the moment"[34]. Many of them used a five-point Likert scale.

SRH in this paper was assessed with a single question: "Please select the item that accurately describes your overall current health". The response item was a five-

For peer review only - http://bmjopen.bmj.com/site/about/guidelines.xhtml

point Likert scale (very good, good, fair, poor, very poor). Guided by common practice [9],[35],[17],[13],[19],[36],[37], this paper classified the responses into a dichotomized dependent variable for a measure of good SRH by combining very good and good health into 1 and 0 otherwise.

Focal Variables: Perceived Environmental Quality

Perceptions of environmental quality (*PEQ*), the main independent variables, consisted of natural and social environmental quality. Natural environmental quality were collected by asking respondents to state the extent of pollutions of air quality, marine resources, other pollutions such as noise, olfactory/odour and others; and social environment was indicated with neighbourhood crime. These variables were measured in different points of Likert scale. "How would you characterize the air quality in your area?" had three scales (1=very clean, 2=fairly clean, and 3=not clean); the last category was treated as the reference group in the logistic model. Two other questions were asked on the importance of air quality improvement, and commitment to it: "How important is air quality improvement to you?" and "Compared to other people, how would you describe your commitment to preserving and improving air quality?". The improvement in air quality was treated as a dichotomous variable with the value of 1 for very important or somewhat important, and 0 for not very important or not important at all. The commitment to improve air quality was treated as a dichotomous variable by giving the value of 1 for much more committed, somewhat more committed, or about the same and 0 for somewhat less committed or much less committed.

For peer review only - http://bmjopen.bmj.com/site/about/guidelines.xhtml

Perception on water supply quality and other pollution were Yes-No questions: "Do you have any problem with water supply?", and "Is the dwelling exposed to noise, odour, or pollution problems?". The answer for "yes" was given the value of 1 and 0 otherwise. "How would you describe the condition of the marine resources (e.g., fisheries, coral reefs etc.) in your area today?" This response was treated as a dichotomous variable by giving the value of 1 for very good, good, or not good not bad, and 0 for bad or very bad. Only one variable was available for social environmental quality. "Do you consider your area a high, medium or low crime area?" It was treated as a dichotomous variable with 1 for medium or high crime and 0 for low crime.

Control Variables

Demographic and Socio-Economic Variables

Control variables consisted of demographic, socio-economic, and healthy lifestyle variable. Demographic variables included age, sex, marital status, and ethnic group. Sex was treated as a dichotomous with the value of 1 for male and 0 otherwise. Age was treated as a numerical variable for the logistic model and was treated as a categorical variable (18-29, 30-59, and 60-85 years old) for Chi-square analysis. Marital status was categorized into three groups, namely, married, ever married and single with married as the reference group. Ethnicity consisted of four groups: Malay, Indigenous Malay, Chinese and Others.

Social-economic variables (*SES*) included education, employment status and ownership of the house. Education had four groups (primary, secondary, post-

For peer review only - http://bmjopen.bmj.com/site/about/guidelines.xhtml

secondary and higher degree). Employment status was categorized into employed, unemployed and not in the labour force (i.e., those who are neither in employment nor in unemployment). Being not in the labour force was treated as the reference group. House ownership was a dichotomous variable (1 for owning the house and 0 for not owning the house).

Healthy Lifestyle

Due to limited information, healthy lifestyle (*HLS*) was measured with survey question asking for the frequency of doing physical activities: "How often do you play sports or exercise per week?" It had four responses: 1=never, 2= 1 to 2 times, 3=3 to 5 times, and 4= everyday. Never play sport or exercise was the reference category.

Statistical Methods

Chi-square statistics was initially performed through a series of cross-tabulations between self-rated health status and all selected variables. Logistic regression model was used for assessing the association between perceived environmental quality and SRH. Below is the logistics regression model used in this paper.

$$\ln\left(\frac{p}{1-p}\right) = \beta_0 + \beta_j PEQ_j + \beta_k DEM_k + \beta_l SES_l + \beta_m HLS_m + \varepsilon$$

where p refers to probability of good self-rate health (SRH)

PEQ refers to perceived environmental quality with j indicators

DEM refers to demographic control variable, with k indicators

SES refers to socio-economic variables with i indicators

HLS refers to healthy lifestyle with only 1 indicator

For peer review only

Following previous study[10], the model was applied in four stages. Model 1 assessed the association between SRH and *PEQ* without control variables. Model 2 enriched Model 1 by adding demographic variables, Model 3 further added *SES* while Model 4 added *HLS* as controls. The *p-value* of 0.05 was selected to determine the significance of the variables. Odds Ratio (OR) and its 95% Confident Intervals (CI) were provided to examine the significance of the relationship.

RESULTS

Sample description

An overview of the characteristics of the respondents based on demographic, socioeconomic variables and environmental quality is presented in Table 1. More than half were female (52.3%) and mature ages between 30-59 years old (51.0%). The majority was married (55.6%), with a significant percentage of single (37.7%). The rest was ever married, either widowed or divorced (6.7%). Brunei Malay accounted for the largest ethnic group (63.3%). More than half attended post-secondary and above (48.5%). About 65% was employed. Less than half did not own the houses they lived in. Regarding the healthy lifestyle, only one fifth never participated in physical exercise, and many of them had their physical activities once or twice per week.

Table 1 about here

Perceived Environmental Quality

Most residents perceived fairly good air quality in their areas (80.2%), very good air quality (12.5%) and perceived air pollution (7.3%). They expressed the need to improve the air quality and half of them were committed to it (Table 2). About 77% did not have problems with sources

For peer review only

of water supply in their daily life. The majority (73.7%) did not perceive the existence of other pollutions such as noise, olfactory and others (Table 2). However, a lower percentage, more than half of them (53.8%) perceived that the condition of marine resources in the district was not good. Social environmental quality was reported frequently by 71.9% of residents considering their areas having low crime.

Table 2 about here

Differential of SRH: Chi-square analysis

Overall, the majority (72.7%) perceived good self-rated health (SRH). The Chi-square analysis presented in Table 2 shows that the prevalence of good SRH significantly differed by perceived air quality (*p-value*=0.002), marine resources (*p-value*=001) and water supply (*p-value*=0.026). The prevalence of good SRH for respondents living in perceived very clean air areas was higher (77.4%) than those living in fairly clean air areas (73.9%), and much higher than those living in polluted or not clean air areas (52.0%). Perceived air quality was thus positively associated with good SRH. However, the prevalence of good SRH was equal between respondents perceiving very importantly to improve air quality and those perceiving not importantly to do so (Table 2). In other words, commitment to improve air quality was not important in good SRH, though perceived air quality was positively related to good SRH.

The prevalence of good SRH was higher among those living in areas perceived to have good marine resources than those living in areas not having good marine resources (78.8% and 67.4%, respectively, *p-value*<0.010). The prevalence of good SRH was equal between respondents having exposure to noise, olfactory and other pollutants and those not being exposed to these pollutants.

For peer review only - http://bmjopen.bmj.com/site/about/guidelines.xhtml

Prevalence of good SRH was higher among those living in a house without any problems on water supply (74.9%) than those having problems with water supply (65.4%, *p-value*<0.005). Social environment condition, measured with perception on safety from crime, was not associated with good SRH.

Age mattered for health status. Table 1 shows that prevalence of good SRH was lower for the older ages than the younger ages. Men and women perceived almost equally in good SRH (72.9% and 72.4%, respectively). There was also no ethnic differential in the prevalence of good SRH (*p-value*=0.349).

Among socio-economic variables, employment status is the only variable which significantly differentiated good SRH (*p-value*=0.002). The prevalence of good SRH for either employed (76.4%) or unemployed (77.3%) was higher than those who were not in the labour force (63.0%). Marital status, education and home ownership were not significantly associated with SRH.

Physical exercise mattered more (p<0.001). Prevalence of good SRH was much higher (88.3%) among respondents exercising regularly between 3 and 5 times per week than those who never doing any physical exercise (63.4%).

Logistic regression of self-rated health

There are four logistic regression results presented in Tables 3 and 4. Model 1 examines the association between perception on environmental quality and SRH, without controlling for other sets of variables. This model shows that respondents with perception of living in fairly clean air areas (OR=2.42, 95% CI 1.30 to 4.49, *p-value*=0.005) or very clean air areas (OR=2.40, 95% CI 1.07 to 5.38, *p-value*=0.034) were

For peer review only - http://bmjopen.bmj.com/site/about/guidelines.xhtml

For peer review only

more likely to state in good SRH than those living in the areas with air pollution. This pattern was slightly different from the Chi-square result with respondents in very clean air areas having the highest prevalence of good SRH.

Table 3 about here

Respondents' opinion and commitment on improving air quality were not associated with SRH (Table 3). Respondents living in areas with perceived good marine resources were more likely to report good SRH than those living in not good condition of marine resources (OR=1.69, 95% CI 1.18 to 2.44, *p-value*=0.005).

However, good health was not significantly associated with respondents' perception on other environmental qualities (water supply; and pollution of noise, olfactory and others). Model 1 changes the significance level of the association between water supply and good SRH into insignificant from the Chi-square result. This indicates that the presence of perception on air quality and marine had more salient influence on the association.

A social environmental condition measured by perception on crime was neither significantly associated with SRH. These findings on the association between perceived environmental quality on good SRH remain the same after controlling for demographic variables (Model 2).

It is worth briefly discussing the findings on demographic variables. Among the selected demographic variables, age was negatively associated with self-rated health (Model 2). The odds of stating good SRH decreased with age (OR=0.98, 95% CI 0.96 to 0.99, *p-value*=0.001). Men and women had the same likelihood of reporting

For peer review only - http://bmjopen.bmj.com/site/about/guidelines.xhtml

good SRH. There was also no difference in the likelihood of reporting good SRH among marital status and ethnic groups.

Adding socio-economic variables, in model 3 (Table 4), all associations between perceived environmental quality and SRH remain the same with a slight change on the magnitude of the perceived air quality and marine resources. The adjusted odds ratios of reporting good SRH was lower in fairly clean air areas (OR=2.35, 95% CI 1.25 to 4.44, *p-value*=0.008) and higher in very clean air areas (OR=2.44, 95% CI 1.06 to 5.63, *p-value*=0.036) than the odds ratios presented in unadjusted model (model 1). In other words, model 3 shows a positive relationship between perceived air quality and good SRH, which was similar to the Chi-square result.

Table 4 about here

The association between perceived good marine resources quality and SRH became stronger (OR=1.77, 95% CI 1.20 to 2.60, *p-value*=0.004 in model 3 compared to OR=1.69 in model 1). In other words, controlling for socio-economic variables, the association between perceived environmental quality (especially air quality and marine resources) and SRH became stronger. Socio-economic variables thus strengthened the relationship.

Among socio-economic variables, employment status was the only variable significantly associated with good SRH. Employed respondents were more likely to report good SRH than non-labour force (OR=1.63, 95% CI 1.05 to 2.53, *p-value*=0.029). This likelihood was not different between unemployed and non-labour force. In addition, the likelihood of reporting good SRH was not significant for educational attainment and home ownership.

For peer review only - http://bmjopen.bmj.com/site/about/guidelines.xhtml

Finally, model 4 shows that healthy lifestyle measured with frequency of doing physical exercise played the most prominent association with SRH. Doing physical exercise three to five times per week was beneficial for good health (OR=3.89, 95% CI 1.96 to 7.71, *p-value*=0.000, the highest odd ratio). However, the health impact of doing physical exercise once or twice, or even everyday weekly was not significantly different from those never doing any exercise.

Interestingly, the inclusion of physical exercise into the model changed the association between perceived air quality and SRH into a weak association (*p-value*=0.059). The association between age and SRH also became insignificant. In other word, physical exercise played a mediating role in the association between perceived environmental quality and good SRH. The inclusion of physical activity strengthened the association between employment status and good SRH as the OR for employed in Model 4 (OR=1.72, 95% CI 1.09 to 2.71, *p-value*=0.021) was higher than Model 3.

DISCUSSION

Environmental variables

This study contributes to the literature by empirically investigate the relationship between perceived environmental quality and SRH for Brunei, a Southeast Asian country with high per capita GDP. Our findings suggest the importance of addressing natural environmental qualities across different dimensions to gauge their differential relationships with SRH. Specifically, the study found that residents who perceived clean air, and good marine resources in their surrounding areas were more likely to report good SRH than residents who perceived polluted air and polluted marine

For peer review only - http://bmjopen.bmj.com/site/about/guidelines.xhtml

resources. Our findings are in line with a previous study that marine quality had a positive relationship with health[38]. Marine and coastal areas have been extensively polluted with various forms of plastics such as water bottles, plastic bags, children' toys, wrappers, medical waste and many others. Plastics affect marine resources through the release of microplastics as harmful pollutant[38]. Polluted marine may pollute the fish and other consumable sea commodities such as seaweed, shellfish and others. This may have further implication to the physical health as many people consume the edible marine resources collected and caught in the sea.

It is therefore important to pay attention to health impact of marine resources pollution as the possibility of both onshore and offshore oil spills to occur in the oil production areas such as Belait district is very high. The health effect of oil spills could last long and residents' exposure to hydrocarbon had detrimental effect on foetal development and infant mortality as shown in the case of Nigeria, an oil producing country [39]. However, the land-based activities such as mining, agriculture, or forestry, industrial activities, household consumption, river-based transportation, and chemical industries can also be a major source of marine pollution[40].

Furthermore, residents who perceived living in clean air areas were more likely to report good health. Our findings are consistent with earlier studies[24], [25], [41]. Poor air quality could cause respiratory infection[27], [42]. Polluted air was also associated with cardiovascular disease, lung cancer, and lung function. As part of Southeast Asian region, Bruneians are prone to forest fires especially during the dry season[43], [44]. Seria, one of the selected sub-district area, was the hotspot area for forest fires[43]. Bushfires have also often become public health concern in other

For peer review only - http://bmjopen.bmj.com/site/about/guidelines.xhtml

For peer review only

countries such as Australia[45], Canada[46] and the US[47]. In addition, exposure to transboundary haze from surrounding ASEAN countries was occasionally experienced in Brunei [26].

Both indoor and outdoor air pollution have been a major concern for global public health[42]. Open burning of household's rubbish is another source of air pollution. Air pollution, measured objectively by particulate molecule (PM_{10}) exceeding the level for good quality of air, in Belait had the longest recorded days among districts[48]. The lessons from various studies around the Ecuadorian Amazon regions was that crude-oil production activities contributed negative health impacts to communities in the form of various cancers such as rectum, skin, kidney, cervix, stomach, skin, leukemia, soft tissue, and cervix[49]. Therefore, policy making needs to consider surrounding environment to improve people health.

At the same time, our findings also indicate that commitment to improve air quality was not important in good SRH, though perceived air quality was positively related to good SRH. Perhaps, the respondents believed that the government could do better in preserving the air quality.

Noise pollution among urban residents of China was positively associated with poor SRH[19],[24]. However, in Brunei case, our finding reveals that perceived olfactory (odour) and noise pollutant was not significantly related to health, although 26.3% of the individuals in the selected sample perceived to be exposed to olfactory and noise pollutants. It should be noted here that the questionnaire recorded information on "olfactory pollutant" and "noise pollutant" in one group.

For peer review only - http://bmjopen.bmj.com/site/about/guidelines.xhtml

The impact of noise on health was mostly from road traffic[50]. Noise from road-traffic may result in nervousness and disturb sleep quality. Nevertheless, as road traffic is relatively low in Brunei in general and in Belait in particular, compared to many other countries, noise pollutant may not have any impact on health in our study. Moreover, literature review[51] revealed that there was limited empirical evidence on the association between olfactory pollutant and health. As such, our finding may indicate that olfactory and noise pollution might be a nuisance but did not necessarily affect health.

Non-environmental variables

Another important finding is that healthy lifestyle such as frequent physical exercise can compensate the health impact of perceived environmental quality. Physical activity mediated the relationship between perceived air quality and SRH. Our findings show that if residents actively and regularly practiced physical exercise, the difference of prevalence in good SRH became insignificant between residents living in perceived clean air and polluted air. In other words, regular physical activity can mitigate the adverse environmental consequences on health. Our findings reveal that regular exercise was positively associated with good SRH, confirmed earlier studies[15], [32], [36].

Previous studies showed that the association between age and health was non-conclusive. Age was not significantly associated with SRH[19]. On the other hand, in our study, age was negatively associated with good SRH when the model was not

For peer review only - http://bmjopen.bmj.com/site/about/guidelines.xhtml

controlled with physical activity. This means that as people became older, the prevalence in good health declined, supporting earlier studies[17], [24], [32], [35].

However, our finding further suggests a mediating effect of physical activity to the relationship between age and SRH which turned this relationship into insignificant. In other words, for the same level of physical activity, age did no longer differentiate SRH. The result indicates that the older people who exercised regularly may have insignificant difference in good health compared to younger people. In other words, physical activity played a very important role on SRH for all ages.

Another important variable associated with good SRH was employment. Previous studies indicated that work-health relationship can be simultaneous. The relationship can be a result of bias which only healthy people are willing to work and unhealthy ones are leaving their jobs. Poor SRH can thus be associated with being unemployed or increasing risk of job loss [52]. On the other hand, the relationship between employment and health had mixed results, being employed can be associated with good SRH, bad SRH or no relationship[52]. Being employed with poorly paid job or stressful jobs can be associated with poor SRH. However, our study found a positive association -- being employed was more likely to be good SRH (OR=1.72, 95% CI 1.09 to 2.71, p-value=0.021) than being out of labour force or unemployed.

Conclusion

Our study concludes that perception on marine resources and air quality mattered to the residents' self-rated health status. Moreover, physical exercise and employment played important roles for health. Therefore, special attention should be spent to

For peer review only

improve air quality and marine resources. Furthermore, there is a strong need for policy intervention to mitigate the negative health impact of environmental quality through promoting physical exercise and employment. Further studies should explore more comprehensive measurements of environmental quality, healthy lifestyle and employment variables in assessing health and environmental quality.

Acknowledgements

The authors would like to thank the Brunei Shell Petroleum (BSP) Sendirian Berhad for providing the funding to conduct this study. The authors also would like to thank the field research team who worked hard to conduct data collection.

Author Contributions. ENA designed the concept, analysed data, chose the method, and wrote the manuscript. C-YH supervised project administration and data collection, wrote, reviewed and edited the final draft. LS coordinated the data collection, reviewed and edited the final draft. AT facilitated funding acquisition, analysed data, reviewed the methods and edited the final draft. All authors contributed to read, edit and approve the final draft of the manuscript.

A Competing interests statement. None declared.

Funding statement. The work was supported by a research grant sponsored by the Brunei Shell Petroleum (BSP) Sendirian Berhad with the reference no.: BSP/SI2018.011/CEA4. The funder had no role in the study design, data collection and analysis, decision to publish, or preparation of the manuscript. The authors independently analysed the anonymous data.

Data statement. No data are available for public due to data agreement.

Patient consent for publication Not required.

Informed consent. Respondents did give informed consent.

Word count: 4699 words

For peer review only - http://bmjopen.bmj.com/site/about/guidelines.xhtml

References

[1] B. A. Craig *et al.*, "The association between self-rated health and social environments, health behaviors and health outcomes: A structural equation analysis," *BMC Public Health*, vol. 18, no. 1, pp. 1–10, 2018, doi: 10.1186/s12889-018-5323-y.

[2] N. Hoeymans, E. J. M. Feskens, D. Kromhout, and G. A. M. Van Den Bos, "The contribution of chronic conditions and disabilities to poor self- rated health in elderly men," *Journals Gerontol. - Ser. A Biol. Sci. Med. Sci.*, vol. 54, no. 10, pp. 501–506, 1999, doi: 10.1093/gerona/54.10.M501.

[3] H. W. Lee, M. Song, J. J. Yang, and D. Kang, "Determinants of poor self-rated health in Korean adults with diabetes," *J. Prev. Med. Public Heal.*, vol. 48, no. 6, pp. 287–300, 2015, doi: 10.3961/jpmph.15.048.

[4] Y. Li *et al.*, "Determinants of self-rated health among an older Tibetan population in a Chinese plateau area: analysis based on the conceptual framework for determinants of health," *BMC Public Health*, vol. 21, no. 1, pp. 1–11, 2021, doi: 10.1186/s12889-021-10359-x.

[5] J. H. Kim, K. R. Kim, K. H. Cho, K. B. Yoo, J. A. Kwon, and E. C. Park, "The association between sleep duration and self-rated health in the Korean general population," *J. Clin. Sleep Med.*, vol. 9, no. 10, pp. 1057–1064, 2013, doi: 10.5664/jcsm.3082.

[6] H. Pikhart, *Social and Psychosocial Determinants of Self-Rated Health in Central and Eastern Europe*. 2002.

[7] M. Wuorela, S. Lavonius, M. Salminen, T. Vahlberg, M. Viitanen, and L.

For peer review only - http://bmjopen.bmj.com/site/about/guidelines.xhtml

Viikari, "Self-rated health and objective health status as predictors of all-cause mortality among older people: A prospective study with a 5-, 10-, and 27-year follow-up," *BMC Geriatr.*, vol. 20, no. 1, pp. 1–7, 2020, doi: 10.1186/s12877-020-01516-9.

[8] P. M. Krueger, K. Dovel, and J. T. Denney, "Democracy and self-rated health across 67 countries: A multilevel analysis," *Soc. Sci. Med.*, vol. 143, pp. 137–144, 2015, doi: 10.1016/j.socscimed.2015.08.047.

[9] H. Chen, Y. Liu, Z. Zhu, and Z. Li, "Does where you live matter to your health? Investigating factors that influence the self-rated health of urban and rural Chinese residents: Evidence drawn from Chinese General Social Survey data," *Health Qual. Life Outcomes*, vol. 15, no. 1, pp. 1–11, 2017, doi: 10.1186/s12955-017-0658-0.

[10] J. H. Park and K. S. Lee, "Self-rated health and its determinants in Japan and South Korea," *Public Health*, vol. 127, no. 9, pp. 834–843, 2013, doi: 10.1016/j.puhe.2012.12.012.

[11] V. Baidin, C. J. Gerry, and M. Kaneva, "How Self-Rated is Self-Rated Health? Exploring the Role of Individual and Institutional Factors in Reporting Heterogeneity in Russia," *Soc. Indic. Res.*, vol. 155, no. 2, 2021, doi: 10.1007/s11205-020-02604-4.

[12] S. Cullati, S. Mukhopadhyay, S. Sieber, A. Chakraborty, and C. Burton-Jeangros, "Is the single self-rated health item reliable in India? A construct validity study," *BMJ Glob. Heal.*, vol. 3, no. 6, pp. 1–12, 2018, doi: 10.1136/bmjgh-2018-000856.

For peer review only - http://bmjopen.bmj.com/site/about/guidelines.xhtml

[13] C. K. Haddock *et al.*, "The validity of self-rated health as a measure of health status among young military personnel: Evidence from a cross-sectional survey," *Health Qual. Life Outcomes*, vol. 4, pp. 1–9, 2006, doi: 10.1186/1477-7525-4-57.

[14] E. L. Idler and Y. Benyamini, "Self-Rated Health and Mortality : A Review of Twenty-Seven Community Studies Author (s): Ellen L . Idler and Yael Benyamini Source : Journal of Health and Social Behavior , Vol . 38 , No . 1 (Mar ., 1997), pp . 21-37 Published by : American Sociologic," *J. Health Soc. Behav.*, vol. 38, no. 1, pp. 21–37, 1997.

[15] H. Jradi, Z. Alharbi, and Y. Mohammad, "Self-rated health among Saudi women: Association with morbidity, lifestyle, and psychosocial factors," *J. Epidemiol. Glob. Health*, vol. 8, no. 3–4, pp. 183–188, 2018, doi: 10.2991/j.jegh.2018.04.100.

[16] T. L. Vie, K. O. Hufthammer, E. Meland, and H. J. Breidablik, "Self-rated health (SRH) in young people and causes of death and mortality in young adulthood. A prospective registry-based Norwegian HUNT-study," *SSM - Popul. Heal.*, vol. 7, no. January, p. 100364, 2019, doi: 10.1016/j.ssmph.2019.100364.

[17] S. Cummins, M. Stafford, S. Macintyre, M. Marmot, and A. Ellaway, "Neighbourhood environment and its association with self rated health: Evidence from Scotland and England," *J. Epidemiol. Community Health*, vol. 59, no. 3, pp. 207–213, 2005, doi: 10.1136/jech.2003.016147.

[18] L. Hale *et al.*, "Perceived neighborhood quality, sleep quality, and health

For peer review only

status: Evidence from the Survey of the Health of Wisconsin," *Soc. Sci. Med.*, vol. 79, no. 1, pp. 16–22, 2013, doi: 10.1016/j.socscimed.2012.07.021.

[19] J. Y. Ou, J. L. Peters, J. I. Levy, R. Bongiovanni, A. Rossini, and M. K. Scammell, "Self-rated health and its association with perceived environmental hazards, the social environment, and cultural stressors in an environmental justice population," *BMC Public Health*, vol. 18, no. 1, pp. 1–10, 2018, doi: 10.1186/s12889-018-5797-7.

[20] K. V. Patel, K. Eschbach, L. L. Rudkin, M. K. Peek, and K. S. Markides, "Neighborhood context and self-rated health in older Mexican Americans," *Ann. Epidemiol.*, vol. 13, no. 9, pp. 620–628, 2003, doi: 10.1016/S1047-2797(03)00060-7.

[21] W. Poortinga, F. D. Dunstan, and D. L. Fone, "Perceptions of the neighbourhood environment and self rated health: A multilevel analysis of the Caerphilly Health and Social Needs Study," *BMC Public Health*, vol. 7, pp. 1–10, 2007, doi: 10.1186/1471-2458-7-285.

[22] S. Roh, Y. Jang, D. A. Chiriboga, K. H. Kwag, S. Cho, and K. Bernstein, "Perceived neighborhood environment affecting physical and mental health: A study with Korean American older adults in New York City," *J. Immigr. Minor. Heal.*, vol. 13, no. 6, pp. 1005–1012, 2011, doi: 10.1007/s10903-011-9492-3.

[23] B. Perera, T. Østbye, and C. Jayawardana, "Neighborhood environment and self-rated health among adults in southern Sri Lanka," *Int. J. Environ. Res. Public Health*, vol. 6, no. 8, pp. 2102–2112, 2009, doi: 10.3390/ijerph6082102.

[24] T. Yang, "Association between perceived environmental pollution and health

For peer review only

For peer review only - http://bmjopen.bmj.com/site/about/guidelines.xhtml

among urban and rural residents-a Chinese national study," *BMC Public Health*, vol. 20, no. 1, pp. 1–10, 2020, doi: 10.1186/s12889-020-8204-0.

[25] J. Liu, Y. Luo, W. Haller, B. Vander Mey, and E. Granberg, "Neighborhood environments and self-rated health in Mainland China, Japan and South Korea," *PLoS One*, vol. 13, no. 9, pp. 1–17, 2018, doi: 10.1371/journal.pone.0204910.

[26] S. Q. Dotse, L. Dagar, M. I. Petra, and L. C. De Silva, "Influence of Southeast Asian Haze episodes on high PM10 concentrations across Brunei Darussalam," *Environ. Pollut.*, vol. 219, pp. 337–352, 2016, doi: 10.1016/j.envpol.2016.10.059.

[27] L. M. T. Luong, P. D. Sly, P. K. Thai, and D. Phung, "Impact of ambient air pollution and wheeze-associated disorders in children in Southeast Asia: A systematic review and meta-analysis," *Rev. Environ. Health*, vol. 34, no. 2, pp. 125–139, 2019, doi: 10.1515/reveh-2018-0079.

[28] B. Bowe, Y. Xie, T. Li, Y. Yan, H. Xian, and Z. Al-Aly, "Estimates of the 2016 global burden of kidney disease attributable to ambient fine particulate matter air pollution," *BMJ Open*, vol. 9, no. 5, 2019, doi: 10.1136/bmjopen-2018-022450.

[29] United Nations, *Human Development Report 2019*. 2019.

[30] E. von Elm, D. G. Altman, M. Egger, S. J. Pocock, P. C. Gøtzsche, and J. P. Vandenbroucke, "The strengthening the reporting of observational studies in epidemiology (STROBE) statement: Guidelines for reporting observational studies," *Int. J. Surg.*, vol. 12, no. 12, pp. 1495–1499, 2014, doi: 10.1016/j.ijsu.2014.07.013.

For peer review only

For peer review only - http://bmjopen.bmj.com/site/about/guidelines.xhtml

[31] B. D. Department of Economic Planning and Statistics, "Report of the Mid-Year Population Estimates 2020," 2021.

[32] A. Rütten *et al.*, "Self reported physical activity, public health, and perceived environment: Results from a comparative European study," *J. Epidemiol. Community Health*, vol. 55, no. 2, pp. 139–146, 2001, doi: 10.1136/jech.55.2.139.

[33] A. K. Harschel, L. A. Schaap, S. Iwarsson, V. Horstmann, and S. Tomsone, "Self-rated health among very old people in European countries: An explorative study in Latvia and Sweden," *Gerontol. Geriatr. Med.*, no. January-December, 2015, doi: 10.1177/2333721415598432.

[34] T. L. Vie, K. O. Hufthammer, T. L. Holmen, E. Meland, and H. J. Breidablik, "Is self-rated health in adolescence a predictor of prescribed medication in adulthood? Findings from the Nord Trøndelag Health Study and the Norwegian Prescription Database," *SSM - Popul. Heal.*, vol. 4, no. November 2017, pp. 144–152, 2018, doi: 10.1016/j.ssmph.2017.11.010.

[35] J. W. Choi, J. Kim, E. Han, and T. H. Kim, "Association of employment status and rated health among income with self- - waged workers with disabilities in South Korea : population- - based panel study," pp. 1–6, 2019, doi: 10.1136/bmjopen-2019-032174.

[36] W. J. Stronegger, S. Titze, and P. Oja, "Perceived characteristics of the neighborhood and its association with physical activity behavior and self-rated health," *Heal. Place*, vol. 16, no. 4, pp. 736–743, 2010, doi: 10.1016/j.healthplace.2010.03.005.

[37] M. Wen, L. C. Hawkley, and J. T. Cacioppo, "Objective and perceived

For peer review only - http://bmjopen.bmj.com/site/about/guidelines.xhtml

neighborhood environment, individual SES and psychosocial factors, and self-rated health: An analysis of older adults in Cook County, Illinois," *Soc. Sci. Med.*, vol. 63, no. 10, pp. 2575–2590, 2006, doi: 10.1016/j.socscimed.2006.06.025.

[38] R. Krishna, M. Mohan, P. Mallika, and D. Costa, "Microplastics in ballast water as an emerging source and vector for harmful chemicals , antibiotics , metals , bacterial pathogens and HAB species : A potential risk to the marine environment and human health," *Mar. Pollut. Bull.*, vol. 149, no. July, p. 110525, 2019, doi: 10.1016/j.marpolbul.2019.110525.

[39] A. Bruederle and R. Hodler, "Effect of oil spills on infant mortality in Nigeria," *Proc. Natl. Acad. Sci. U. S. A.*, vol. 116, no. 12, pp. 5467–5471, 2019, doi: 10.1073/pnas.1818303116.

[40] K. A. Willis *et al.*, "Cleaner seas: reducing marine pollution," *Rev. Fish Biol. Fish.*, vol. 8, 2021, doi: 10.1007/s11160-021-09674-8.

[41] P. Liu, J. Wang, X. Wang, W. Nie, and F. Zhen, "Measuring the association of self-perceived physical and social neighborhood environment with health of Chinese rural residents," *Int. J. Environ. Res. Public Health*, vol. 18, no. 16, pp. 1–12, 2021, doi: 10.3390/ijerph18168380.

[42] N. Khaltaev and S. Axelrod, "Chronic respiratory diseases global mortality trends, treatment guidelines, life style modifications, and air pollution: Preliminary analysis," *J. Thorac. Dis.*, vol. 11, no. 6, pp. 2643–2655, 2019, doi: 10.21037/jtd.2019.06.08.

[43] M. Radojevic and H. Hassan, "Air quality in Brunei Darussalam during the 1998 haze episode," *Atmos. Environ.*, vol. 33, no. 22, pp. 3651–3658, 1999, doi:

For peer review only - http://bmjopen.bmj.com/site/about/guidelines.xhtml

10.1016/S1352-2310(99)00118-1.

[44] S. Shahriar, Z. El-Said M. M., S. Safwanah N. M., H. Kho J., N. N. D. H. M. F. Lee, and N. H. H. Hasim, “Risk assessment for forest fire in Brunei Darussalam,” *MATEC Web Conf.*, vol. 258, p. 05033, 2019, doi: 10.1051/matecconf/201925805033.

[45] N. Borchers Arriagada, A. J. Palmer, D. M. J. S. Bowman, G. G. Morgan, B. B. Jalaludin, and F. H. Johnston, “Unprecedented smoke-related health burden associated with the 2019–20 bushfires in eastern Australia,” *Med. J. Aust.*, vol. 213, no. 6, pp. 282–283, 2020, doi: 10.5694/mja2.50545.

[46] C. J. Matz *et al.*, “Health impact analysis of PM2.5 from wildfire smoke in Canada (2013–2015, 2017–2018),” *Sci. Total Environ.*, vol. 725, p. 138506, 2020, doi: 10.1016/j.scitotenv.2020.138506.

[47] N. Fann *et al.*, “The health impacts and economic value of wildland fire episodes in the U.S.: 2008–2012,” *Sci. Total Environ.*, vol. 610–611, pp. 802–809, 2018, doi: 10.1016/j.scitotenv.2017.08.024.

[48] E. N. Aziz, A. Kasem, W. S. Haji Suhaili, and P. Zhao, “Convolution recurrent neural network for daily forecast of PM10 concentrations in Brunei Darussalam,” *Chem. Eng. Trans.*, vol. 83, no. February, pp. 355–360, 2021, doi: 10.3303/CET2183060.

[49] G. Coronel Vargas, W. W. Au, and A. Izzotti, “Public health issues from crude-oil production in the Ecuadorian Amazon territories,” *Sci. Total Environ.*, vol. 719, p. 134647, 2020, doi: 10.1016/j.scitotenv.2019.134647.

[50] S. Geravandi *et al.*, “Noise Pollution and Health Effects,” *Jundishapur J. Heal.*

For peer review only

For peer review only - http://bmjopen.bmj.com/site/about/guidelines.xhtml

Sci., vol. 7, no. 1, pp. 1–15, 2015, doi: 10.5812/jjhs.25357.

[51] V. Guadalupe-Fernandez *et al.*, "Industrial odour pollution and human health: a systematic review and meta-analysis," *Environ. Heal. A Glob. Access Sci. Source*, vol. 20, no. 1, pp. 1–21, 2021, doi: 10.1186/s12940-021-00774-3.

[52] L. Antonisse and R. Garfield, "The Relationship Between Work and Health: Findings from a Literature Review," *Issue Br.*, no. August, 2018, [Online]. Available: https://www.kff.org/medicaid/issue-brief/the-relationship-between-work-and-health-findings-from-a-literature-review/.

For peer review only

For peer review only - http://bmjopen.bmj.com/site/about/guidelines.xhtml

Table 1. Respondents Characteristics and Comparison in Good SRH

Variable		Frequency	Percent	Good SRH	X^2 *p-value*
	Total	673	100.0	-	
Self-rated health	Not good	184	27.3	-	
	Good health	489	72.7	-	
Sex	Female	352	52.3	72.4	0.964
	Male	321	47.7	72.9	
Age	18-29	241	35.8	77.2	0.020
	30-59	343	51.0	72.3	
	60-85	89	13.2	61.8	
Marital status	Married	374	55.6	71.9	0.293
	Ever married	45	6.7	64.4	
	Single	254	37.7	75.2	
Ethnic groups	Brunei Malay	426	63.3	74.9	0.349
	Indigenous Malay	116	17.2	70.7	
	Chinese	75	11.1	66.7	
	Others	56	8.3	67.9	
Education	Primary and below	37	5.5	54.1	0.074
	Secondary	242	36.0	74.0	
	Post-secondary & diploma	221	32.8	74.2	
	High degree	173	25.7	72.8	
House ownership	Own house	304	45.2	70.1	0.171
	Not own	369	54.8	74.8	
Employment status	Employed	437	64.9	76.4	0.002
	Unemployed	44	6.5	77.3	
	Not labour force	192	28.5	63.0	
Physical exercise	Never	142	21.1	63.4	0.000
	1-2 times	362	53.8	70.4	
	3-5 times	128	19.0	88.3	
	Everyday	41	6.1	75.6	

For peer review only - http://bmjopen.bmj.com/site/about/guidelines.xhtml

Table 2. Differential of Good SRH by Perceived Environmental Quality

		Frequency	Percent	Good SRH	X^2 *p-value*
Air quality	Very clean	84	12.5	77.4	
	Fairly clean	540	80.2	73.9	0.002
	Not clean	49	7.3	51.0	
Water problem	No problem	517	76.8	74.9	0.026
	Water supply problem	156	23.2	65.4	
Marine	Not good	362	53.8	67.4	0.001
	Good	311	46.2	78.8	
Other pollutions	Not exposed	496	73.7	74.0	0.230
	Exposed to noise, odour, pollution problems	177	26.3	68.9	
Crime	Low crime	484	71.9	73.3	0.586
	Medium & high	189	28.1	70.9	
Air quality improvement	Not very/not important	169	25.1	71.6	0.796
	Important to improve air quality	504	74.9	73.0	
Commitment	Less committed	313	46.5	72.2	0.873
	Committed	360	53.5	73.1	

For peer review only

For peer review only - http://bmjopen.bmj.com/site/about/guidelines.xhtml

Table 3. Odds Ratios for Good SRH and Perceived Environmental Quality controlled for Demographic Variables

Variables	Model 1			Model 2		
	OR	95% CI	Sig.	OR	95% CI	Sig.
**Perceived environmental**						
Air quality (ref=Not clean)			0.020			0.024
Very clean	2.40	1.07 to 5.38	0.034	2.37	1.04 to 5.41	0.040
Fairly clean	2.42	1.30 to 4.49	0.005	2.39	1.28 to 4.49	0.007
Marine Resources (ref=Not good)						
Good Marine Resources	1.69	1.18 to 2.44	0.005	1.78	1.22 to 2.60	0.003
Other pollutions (ref=Not expose)						
Exposed Noise, odour, & others	1.03	0.68 to 1.55	0.907	1.02	0.68 to 1.56	0.908
Water supply (ref=No problem)						
Problem with water supply	0.71	0.47 to 1.08	0.110	0.74	0.49 to 1.13	0.166
Crime (ref=Low crime)						
Medium and High crime	0.98	0.67 to 1.45	0.933	0.90	0.61 to 1.34	0.610
Air Quality Improvement (ref=Not important)						
Important	0.96	0.64 to 1.45	0.857	0.96	0.63 to 1.45	0.830
Commitment to Air Quality Improvement (ref=Not committed)						
Committed	1.08	0.75 to 1.54	0.687	1.02	0.71 to 1.46	0.934
**Demographic variables**						
Age (in years)	-	-	-	0.98	0.96 to 0.99	0.001
Sex (ref=Female)						
Male	-	-	-	1.02	0.71 to 1.46	0.913
Ethnic group (ref=Brunei Malay)						0.588
Indigenous Malay	-	-	-	0.82	0.51 to 1.31	0.397
Chinese	-	-	-	0.86	0.49 to 1.51	0.597
Others	-	-	-	0.68	0.37 to 1.28	0.231
Marital status (ref=Married)						0.409
Ever married	-	-	-	0.86	0.42 to 1.74	0.669
Single	-	-	-	0.72	0.44 to 1.19	0.201
Constant	1.02		0.967	3.70		0.018

- Ref = reference group

For peer review only - http://bmjopen.bmj.com/site/about/guidelines.xhtml

Table 4. Odds Ratios for Good SRH and Perceived Environmental Quality controlled for Demographic-Socio-Economic and Healthy Lifestyle Variables

Variables	Model 3			Model 4		
	OR	95% CI	Sig.	OR	95% CI	Sig.
**Perceived environmental**						
Air quality (ref=Not clean)			0.029			0.059
Very clean	2.44	1.06 to 5.63	0.036	2.19	0.93 to 5.12	0.072
Fairly clean	2.35	1.25 to 4.44	0.008	2.20	1.15 to 4.22	0.018
Marine Resources (ref=Not good)						
Good Marine Resources	1.77	1.20 to 2.60	0.004	1.84	1.24 to 2.74	0.002
Other pollutions (ref=Not expose)						
Exposed Noise, odour, & others	1.07	0.70 to 1.63	0.771	0.98	0.63 to 1.51	0.911
Water supply (ref=No problem)						
Problem with water supply	0.72	0.47 to 1.11	0.136	0.71	0.46 to 1.10	0.123
Crime (ref=Low crime)						
Medium and High crime	0.90	0.60 to 1.34	0.595	0.97	0.64 to 1.45	0.866
Air Quality Improvement (ref=Not important)						
Important	0.97	0.64 to 1.47	0.874	0.98	0.64 to 1.50	0.926
Commitment to Air Quality Improvement (ref=Not committed)						
Committed	0.99	0.69 to 1.44	0.975	0.92	0.63 to 1.35	0.681
**Demographic variables**						
Age (in years)	0.98	0.96 to 0.99	0.033	0.98	0.97 to 1.00	0.079
Sex (ref=Female)						
Male	0.98	0.68 to 1.42	0.912	0.86	0.59 to 1.26	0.433
Ethnic group (ref=Brunei Malay)			0.599			0.384
Indigenous Malay	0.78	0.49 to 1.26	0.314	0.78	0.48 to 1.26	0.303
Chinese	0.87	0.49 to 1.54	0.632	0.83	0.46 to 1.48	0.519
Others	0.71	0.37 to 1.34	0.287	0.60	0.31 to 1.16	0.126
Marital status (ref=Married)	-		0.585	-		0.552
Ever married	0.89	0.42 to 1.86	0.747	0.77	0.36 to 1.63	0.487
Single	0.77	0.46 to 1.29	0.320	0.79	0.47 to 1.34	0.384
**Socio-economic variables**						
Education (ref=Primary and below)			0.550			0.435
Secondary	1.35	0.60 to 2.99	0.468	1.27	0.56 to 2.88	0.567
Post-secondary and Diploma	1.04	0.438 to 2.46	0.934	0.93	0.39 to 2.26	0.876
Higher education	0.99	0.411to 2.38	0.981	0.87	0.35 to 2.14	0.755
Employment (ref=Not labour force)			0.091			0.068
Employed	1.63	1.05 to 2.53	0.029	1.72	1.09 to 2.71	0.021
Unemployed	1.53	0.66 to 3.57	0.323	1.63	0.68 to 3.89	0.270
House ownership (ref=Not own)						
Own	1.09	0.73 to 1.63	0.669	1.11	0.60 to 1.36	0.626
**Healthy lifestyle**						
Physical activities (ref=Never)						0.000
1-2 times	-	-	-	1.06	0.67 to 1.68	0.795
3-5 times	-	-	-	3.89	1.96 to 7.71	0.000
Everyday	-	-	-	1.94	0.83 to 4.50	0.124

For peer review only - http://bmjopen.bmj.com/site/about/guidelines.xhtml

Constant	1.76		0.466	1.47		0.633

For peer review only

For peer review only - http://bmjopen.bmj.com/site/about/guidelines.xhtml

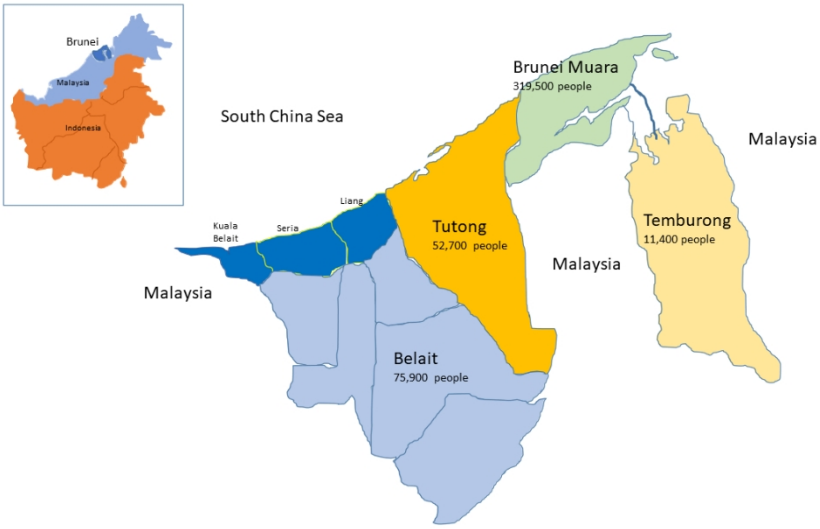

Figure 1. Geographical location of the studied areas

338x190mm (96 x 96 DPI)

Reporting checklist for cross sectional study.

Based on the STROBE cross sectional guidelines.

Instructions to authors

Complete this checklist by entering the page numbers from your manuscript where readers will find each of the items listed below.

Your article may not currently address all the items on the checklist. Please modify your text to include the missing information. If you are certain that an item does not apply, please write "n/a" and provide a short explanation.

Upload your completed checklist as an extra file when you submit to a journal.

In your methods section, say that you used the STROBE cross sectionalreporting guidelines, and cite them as:

von Elm E, Altman DG, Egger M, Pocock SJ, Gotzsche PC, Vandenbroucke JP. The Strengthening the Reporting of Observational Studies in Epidemiology (STROBE) Statement: guidelines for reporting observational studies.

		Reporting Item	Page Number
**Title and abstract**			
Title	#1a	Indicate the study's design with a commonly used term in the title or the abstract	1
Abstract	#1b	Provide in the abstract an informative and balanced summary	1-2

For peer review only

		of what was done and what was found	
**Introduction**			
Background / rationale	#2	Explain the scientific background and rationale for the investigation being reported	3-6
Objectives	#3	State specific objectives, including any prespecified hypotheses	6
**Methods**			
Study design	#4	Present key elements of study design early in the paper	7
Setting	#5	Describe the setting, locations, and relevant dates, including periods of recruitment, exposure, follow-up, and data collection	7
Eligibility criteria	#6a	Give the eligibility criteria, and the sources and methods of selection of participants.	7
	#7	Clearly define all outcomes, exposures, predictors, potential confounders, and effect modifiers. Give diagnostic criteria, if applicable	8-11
Data sources / measurement	#8	For each variable of interest give sources of data and details of methods of assessment (measurement). Describe comparability of assessment methods if there is more than one group. Give information separately for for exposed and unexposed groups if applicable.	8-11
Bias	#9	Describe any efforts to address potential sources of bias	7-8
Study size	#10	Explain how the study size was arrived at	7

For peer review only

Quantitative variables	#11	Explain how quantitative variables were handled in the analyses. If applicable, describe which groupings were chosen, and why	10-11
Statistical methods	#12a	Describe all statistical methods, including those used to control for confounding	10-11
Statistical methods	#12b	Describe any methods used to examine subgroups and interactions	10-11
Statistical methods	#12c	Explain how missing data were addressed	7
Statistical methods	#12d	If applicable, describe analytical methods taking account of sampling strategy	-
Statistical methods	#12e	Describe any sensitivity analyses	-
**Results**			
Participants	#13a	Report numbers of individuals at each stage of study—eg numbers potentially eligible, examined for eligibility, confirmed eligible, included in the study, completing follow-up, and analysed. Give information separately for for exposed and unexposed groups if applicable.	12
Participants	#13b	Give reasons for non-participation at each stage	12
Participants	#13c	Consider use of a flow diagram	
Descriptive data	#14a	Give characteristics of study participants (eg demographic,	12

For peer review only

		clinical, social) and information on exposures and potential confounders. Give information separately for exposed and unexposed groups if applicable.	
Descriptive data	#14b	Indicate number of participants with missing data for each variable of interest	12
Outcome data	#15	Report numbers of outcome events or summary measures. Give information separately for exposed and unexposed groups if applicable.	12
Main results	#16a	Give unadjusted estimates and, if applicable, confounder-adjusted estimates and their precision (eg, 95% confidence interval). Make clear which confounders were adjusted for and why they were included	12-21
Main results	#16b	Report category boundaries when continuous variables were categorized	12-21
Main results	#16c	If relevant, consider translating estimates of relative risk into absolute risk for a meaningful time period	12-21
Other analyses	#17	Report other analyses done—e.g., analyses of subgroups and interactions, and sensitivity analyses	
**Discussion**			
Key results	#18	Summarise key results with reference to study objectives	18-22
Limitations	#19	Discuss limitations of the study, taking into account sources of potential bias or imprecision. Discuss both direction and magnitude of any potential bias.	18-22

For peer review only

Interpretation	#20	Give a cautious overall interpretation considering objectives, limitations, multiplicity of analyses, results from similar studies, and other relevant evidence.	18-22
Generalisability	#21	Discuss the generalisability (external validity) of the study results	22

Other Information

Funding	#22	Give the source of funding and the role of the funders for the present study and, if applicable, for the original study on which the present article is based	2

None The STROBE checklist is distributed under the terms of the Creative Commons Attribution License CC-BY. This checklist can be completed online using https://www.goodreports.org/, a tool made by the EQUATOR Network in collaboration with Penelope.ai

For peer review only

BMJ Open

Self-rated Health and Perceived Environmental Quality in Brunei Darussalam:A cross-sectional study

Journal:	*BMJ Open*
Manuscript ID	bmjopen-2022-060799.R1
Article Type:	Original research
Date Submitted by the Author:	18-May-2022
Complete List of Authors:	Arifin, Evi Nurvidya; Universiti Brunei Darussalam, Centre for Advanced Research (CARe)<br>Hoon, Chang-Yau; Universiti Brunei Darussalam, Centre for Advanced Research (CARe)<br>Slesman, Ly; Universiti Brunei Darussalam, Centre for Advanced Research (CARe)<br>Tan, Abby; Universiti Brunei Darussalam, Chancellery
**Primary Subject Heading**:	Epidemiology
Secondary Subject Heading:	Health policy, Public health
Keywords:	EPIDEMIOLOGY, PUBLIC HEALTH, PRIMARY CARE

SCHOLARONE™
Manuscripts

I, the Submitting Author has the right to grant and does grant on behalf of all authors of the Work (as defined in the below author licence), an exclusive licence and/or a non-exclusive licence for contributions from authors who are: i) UK Crown employees; ii) where BMJ has agreed a CC-BY licence shall apply, and/or iii) in accordance with the terms applicable for US Federal Government officers or employees acting as part of their official duties; on a worldwide, perpetual, irrevocable, royalty-free basis to BMJ Publishing Group Ltd ("BMJ") its licensees and where the relevant Journal is co-owned by BMJ to the co-owners of the Journal, to publish the Work in this journal and any other BMJ products and to exploit all rights, as set out in our licence.

The Submitting Author accepts and understands that any supply made under these terms is made by BMJ to the Submitting Author unless you are acting as an employee on behalf of your employer or a postgraduate student of an affiliated institution which is paying any applicable article publishing charge ("APC") for Open Access articles. Where the Submitting Author wishes to make the Work available on an Open Access basis (and intends to pay the relevant APC), the terms of reuse of such Open Access shall be governed by a Creative Commons licence – details of these licences and which Creative Commons licence will apply to this Work are set out in our licence referred to above.

Other than as permitted in any relevant BMJ Author's Self Archiving Policies, I confirm this Work has not been accepted for publication elsewhere, is not being considered for publication elsewhere and does not duplicate material already published. I confirm all authors consent to publication of this Work and authorise the granting of this licence.

For peer review only

Self-rated Health and Perceived Environmental Quality in Brunei Darussalam: A cross-sectional study

Evi Nurvidya Arifin*
Centre for Advanced Research (CARe), Universiti Brunei Darussalam,
Jalan Tungku Link, Gadong BE1410, Brunei Darussalam
Email: evi.arifin@ubd.edu.bn

Chang-Yau Hoon
Centre for Advanced Research (CARe), Universiti Brunei Darussalam,
Jalan Tungku Link, Gadong BE1410, Brunei Darussalam
Email: changyau.hoon@ubd.edu.bn

Ly Slesman
Centre for Advanced Research (CARe), Universiti Brunei Darussalam,
Jalan Tungku Link, Gadong BE1410, Brunei Darussalam
Email: slesman.ly@ubd.edu.bn

Abby Tan
Chancellery, Universiti Brunei Darussalam,
Jalan Tungku Link, Gadong BE1410, Brunei Darussalam
Email: abby.tan@ubd.edu.bn

Corresponding author: Evi Nurvidya Arifin Centre for Advanced Research (CARe), Universiti Brunei Darussalam, Jalan Tungku Link, Gadong BE1410, Brunei Darussalam. Email address: evi.arifin@ubd.edu.bn. Phone: +673 246 0922/3 Ext 2741.

For peer review only

ABSTRACT

Objectives: This paper examines the relationship between individuals' perceptions of environmental quality and self-rated health (SRH) after controlling for dimensions of socio-economic-demographic and healthy lifestyle variables.
Design: A cross-sectional survey.
Setting: The survey was conducted in Belait, an oil- and gas-rich district, Brunei Darussalam, from 17 October to 11 November 2019. It focuses on the most populated subdistrict (Kuala Belait, Seria, and Liang), where 97% of residents reside.
Participants: A final of 1000 respondents aged 18 years and older were randomly selected from the chosen sub-districts population with a 95% confidence interval and ±3 margin of error. Due to the variable selection, only 673 respondents were available for the analysis.
Outcome measures: SRH was dichotomized with 1 for good health and 0 otherwise. Perception of environmental quality included perceptions of the natural environment (air quality, marine quality, water supply, noise and olfactory pollution) and social

environment (crime). Chi-square and logistic regression models were used to assess the relationship between individuals' perceived environmental quality and SRH.
Results: Most respondents perceived themselves in good SRH (72%). The adjusted logistic regression shows that the perception of air quality (OR=2.20, 95% CI 1.15-4.22, p=0.018) and marine resources (OR=1.84, 95% CI 1.24-2.74, =0.002) in their surrounding areas were significantly associated with good SRH. However, other environmental variables were insignificantly associated with SRH. Among control variables, healthy lifestyle and employment had positive associations with good SRH (OR=3.89, 95% CI 1.96-7.71, p=0.000 for exercising 3-5 times a week, and OR=1.72, 95% CI 1.09-2.71, p=0.021 for being employed). In addition, frequent physical exercise compensated for the negative health impact of environmental pollution.
Conclusions: This study suggests that environmental quality has an important role in SRH. However, a healthy lifestyle measured with the frequency of physical exercise seems to compensate for the adverse environmental effects on SRH.

KEYWORDS: Self-rated health; perceived environmental quality; socio-economic-demographic condition; healthy lifestyle; Brunei Darussalam.

Article Summary

Strengths and limitations of this study

* This study's strength is in providing new empirical evidence on how environmental quality is related to self-rated health, a subjective measure, especially in an oil- and gas-rich Belait district of Brunei Darussalam, one of the Southeast Asian countries.
* Another strength is that it used a representative and a relatively large district sample.
* One limitation is that there is no comparable measure of objective health.
* Perceptions of olfactory and noise pollutants were grouped into one category, and there was only one variable of social environment, therefore, further studies should collect these pollutants and more variables of social environment separately.
* A longitudinal study is important to further examine a causal relationship between perception of environmental quality and health and investigate the dynamics of the relationship.

For peer review only

INTRODUCTION

Ensuring healthy life is Goal 3 of the 17 sustainable development goals[1]. Health crisis has far-reaching consequences on individuals, families, communities and states. At the individual level, being unhealthy encompasses suffering, deprivation of self-confidence and capability to function, and losing productivity and various opportunities. To some extent, being unhealthy can lead to a premature end of life. What happens to individuals may impact the whole family. Unhealthy or sick family members may lead to a family caregiving crisis and disrupt other family members' social and economic activities. They may even fall into poverty[2–4].

Communicable diseases, for example, may even become a pandemic, resulting in a sudden considerable disruption of people's life. At the state level, a health crisis can weaken the heath-care system and destabilize the economy through a contraction in employment, increased state expenditure, and reduced investment. The current COVID-19 pandemic provided evidence of the health crisis' impact on both health and the economy in many countries around the globe[5–7].

To be healthy has multi-dimensional factors[8] with complex roles at different levels (individual, families, community and state). Previous studies were mostly concerned with health-related individual and family factors such as medical, biological or hereditary conditions; psychosocial, demographic, social and economic conditions, and healthy lifestyle[9–14]. State-level health status factors were only considered when the analyses were concerned with cross-countries differences. For example, level of democratic governance was found to have a positive association with health[15]. Yet, studies within a country or region should also consider the health-

For peer review only - http://bmjopen.bmj.com/site/about/guidelines.xhtml

related community factors, especially the importance of surrounding environmental conditions. This environmental condition can take three forms: built or man-made, natural and social environment[16].

Because it is not easy to measure objective measures of environmental quality at the individual level, this paper utilized its proxy -- perceived environmental quality. Most importantly, objective measures of environmental quality apply to all individuals living in the same environment. Then there is no variation among individuals. Thus, the environment-health relationship does not exist in a small study area. The use of subjective measures of environmental quality can vary from one individual to another. Perception of environmental quality may matter to individuals. Individuals' perception of their environmental quality may say more about a variation in the perceptions of environment and health. This study contributes to new empirical findings. This subjective variable was then used to examine the association between the environmental quality of the surrounding areas and self-rated health status. Similarly, on population-based surveys, health status was assessed subjectively, known as self-rated health (SRH). SRH has been widely used in many countries. SRH is a reliable health indicator used in population-based studies[17,18] and provides a reliable and acceptable approximation of the overall health outcomes of the population[18–20]. It captures the biological, mental, and social states of an individual health[21] and has been shown to be able to explain mortality and morbidity[21,22], and causes of deaths[14,23].

Assessing the association between these two subjective domains has been conducted mostly in Western countries[24–30], and some Asian countries such as Sri

For peer review only

For peer review only - http://bmjopen.bmj.com/site/about/guidelines.xhtml

Lanka[31], China[16,32], Japan and South Korea[33]. However, little is known about this relationship among Southeast Asian countries, where countries in this region prospered and developed significantly. Natural environmental issues in this region such as seasonal forest fires, have also been a transboundary concern[34].

Environment is a broad concept and has been measured differently from one study to another. For example, a study in England and Scotland[24] measured neighbourhood environmental conditions related to man-made and social environments such as access to private transportation, physical quality of the residential environment, political climate and political engagement. Similarly, another study in the United Kingdom[28] used these environmental conditions, but the selected variables referred to access to amenities, neighbourhood quality, and neighbourhood disorder. Unlike these studies[24,28], environmental condition among residents of Chelsea, Massachusetts, in the US was measured differently by asking about the perception of the natural environment regarding air quality, odour and noise, and social environment, including feeling safe, neighbourhood crime, and social cohesion[26]. A study in Sri Lanka[31] examined environmental factors using ventilation problems, water shortage, garbage disposal problems, mosquito threat, stray dogs, and social environment (nuisance from neighbours and overcrowding). A study in China concerning its rapid development examined people's perception of air pollution, water pollution, freshwater resource shortage and green space shortage in relation to self-related health[16].

However, findings from the abovementioned studies were inconclusive. The study in England and Scotland found that most of their selected neighbourhood

For peer review only

environmental factors did not associate with SRH[24]. Similarly, none of the environmental factors was associated with SRH[31]. In contrast, some studies found that people's poor living environment was likely associated with a higher likelihood of reporting poor SRH[25–29,33]. Urban residents in China who perceived air pollution and noise pollution were more likely to report not in good SRH[32]. Air pollution had health impacts on respiratory diseases in some Southeast Asian countries[35] and non-communicable diseases such as chronic kidney diseases in 194 countries and territories[36]. Different results were also found in China[32] that perceived environmental pollution was not significantly associated with SRH among rural residents of China. Nevertheless, there has been an insufficient number of empirical studies which assessed the impacts of perceived environmental quality on self-rated health among Southeast Asian countries.

With this background, identifying environmental factors on health is crucial to contribute new empirical findings to the debate on the environment-health relationship, more specifically between perceived environmental quality and self-rated health (SRH). A study in China[16] reported that the advantage of examining subjective indicators than objective indicators on environment-health relationships may provide more stories about individuals' life. This study aims to fill in the gap in this relationship by controlling for demographic, socio-economic and lifestyle variables in Brunei Darussalam (hereafter, Brunei). This study does not consider variables measuring built environmental quality[16] due to the unavailability of information. Our primary objectives are, therefore to estimate the prevalence of good SRH and examine the relationship between natural and social environmental qualities

For peer review only - http://bmjopen.bmj.com/site/about/guidelines.xhtml

and SRH accounting for dimensions of socio-economic-demographic and healthy lifestyle variables based on a cross-sectional study. Brunei's development has led to rapid urbanization transforming its green nature and social environment for human needs.

Brunei has also provided its people with high quality of life, as indicated by its high HDI (Human Development Index), which stood at the 43th worldwide in 2018[37]. Its economy is heavily dependent on oil and gas production, with oil and natural gas exports as its major export and its main source of development and wealth since the mid-20th century. Brunei benefits considerably from the world market oil price, making it the second-highest per capita GDP after Singapore among Southeast Asian countries.

METHODS

Design, Setting, Study size and Participants

We used the Strengthening the Reporting of Observational Studies in Epidemiology (STROBE) cross-sectional checklist when writing our report[38]. The paper used an original cross-sectional survey conducted by the Universiti Brunei Darussalam between 17 October and 11 November 2019, before the COVID-19 pandemic spread to this country in March 2020. The survey was conducted in the Belait district, the largest one of the four districts in Brunei in terms of land size. This district was home to around 75,900 persons or 16.5% of 459,500 Brunei's population in 2019[39]. Within this district, three sub-districts were selected, namely Seria, Liang, and Kuala Belait, where the majority of people (97%) reside and the primary sources of the country's economy

For peer review only - http://bmjopen.bmj.com/site/about/guidelines.xhtml

– the production of hydrocarbon resources - are located. They are located in the northern coastal area facing the South China Sea, as seen in Figure 1.

Figure 1 about here

This survey interviewed 1,000 randomly selected respondents aged 18 years and above residing in the chosen subdistrict (or *Mukim*). Based on the 2016 population census, the population aged 18 years and above in Belait was roughly 72 percent data[40]. This sample size was randomly drawn with a 95% confidence level and ±3% margin of error. Face-to-face interviews were performed by 11 trained research assistants. The survey gathered comprehensive information using a pre-tested structured questionnaire covering background characteristics, employment and income, housing, education and aspirations, health, and environmental impact and awareness. The questions were mostly closed-ended, combined with several open-ended ones. Prior to the interviews, respondents were aware and informed about the survey's objectives. The response for each selected variable varied for various reasons such as do not want to answer, do not know, unclear reason and others. For consistency, 673 respondents who completely responded to all questions were selected for this paper. Table 1 below provides further details of the basic characteristics of the sample.

Patient and Public Involvement

Informed consent was provided for which respondents signed a consent form to agree or not to participate voluntarily.

For peer review only - http://bmjopen.bmj.com/site/about/guidelines.xhtml

Research Ethics Approval

The University Research Ethics Committee at Universiti Brunei Darussalam approved its research ethic with the reference number UBD/OAVCR/UREC/Sep19-04.

Variables and Measurements

The Dependent Variable: Self-rated Health (SRH)

Self-rated health (SRH) has been widely used in population-based surveys due to its simplicity as a singular question. Usually, the question took a form such as "In general, would you say that your health is …?[41–43]. The Likert scale was usually used for the response. However, in practice, the question varies slightly in different contexts. For instance, "Would you say your overall physical health is…?" was used by a study in the US [20]. Studies in Japan and Korea used "How would you rate your health?"[17], and in Norway "How is your health at the moment"[44]. In Timor Leste and India, self-rated physical health was measured by more than one variable and therefore expressed as an index[43,45]. Most of these studies used a Likert scale.

SRH in this paper was assessed with a single question: "Please select the item that accurately describes your overall current health". The response item was a five-point Likert scale (very good, good, fair, poor, very poor). Guided by common practice [16,20,24,26,46–48], this paper classified the responses into a dichotomized dependent variable to measure good SRH by combining very good and good health into 1 and 0 otherwise.

For peer review only

Focal Variables: Perceived Environmental Quality

Perceptions of environmental quality (*PEQ*), the main independent variables, consisted of natural and social environmental quality. Data on natural environmental quality were collected by asking respondents to state the extent of pollution of air quality, marine resources, other pollutions such as noise, olfactory/odour and others; and social environment was indicated with neighbourhood crime. These variables were measured at different points of the Likert scale. "How would you characterize the air quality in your area?" had three scales (1=very clean, 2=fairly clean, and 3=not clean); the last category was treated as the reference group in the logistic model. Two other questions were asked on the importance of air quality improvement, and commitment to it: "How important is air quality improvement to you?" and "Compared to other people, how would you describe your commitment to preserving and improving air quality?". The improvement in air quality was treated as a dichotomous variable with the value of 1 for very important or somewhat important and 0 for not very important or not important at all. The commitment to improve air quality was treated as a dichotomous variable by giving the value of 1 for much more committed, somewhat more committed, or about the same and 0 for somewhat less committed or much less committed.

Perception on water supply quality and other pollution were Yes-No questions: "Do you have any problem with water supply?" and "Is the dwelling exposed to noise, odour, or pollution problems?". The answer for "yes" was given the value of 1

For peer review only - http://bmjopen.bmj.com/site/about/guidelines.xhtml

and 0 otherwise. "How would you describe the condition of the marine resources (e.g., fisheries, coral reefs etc.) in your area today?" This response was treated as a dichotomous variable by giving the value of 1 for very good, good, or not good not bad, and 0 for bad or very bad. Only one variable was available for social environmental quality. "Do you consider your area a high, medium or low crime area?" It was treated as a dichotomous variable with 1 for medium or high crime and 0 for low crime.

For peer review only

Control Variables

Demographic and Socio-Economic Variables

Control variables consisted of demographic, socio-economic, and healthy lifestyle variables. Demographic variables included age, sex, marital status, and ethnic group. Sex was treated as a dichotomous with the value of 1 for males and 0 for females. Age was treated as a numerical variable for the logistic model and a categorical variable (18-29, 30-59, and 60-85 years old) for Chi-square analysis. Marital status was categorized into three groups: married, ever married (divorced and widowed) and single/never married, with married as the reference group. Ethnicity consisted of four groups: Brunei Malay, Indigenous Malay, Chinese and Others. As of the Brunei Nationality Act 1961, the Malay group consists of seven ethnic groups: Brunei Malay or Brunei, Belait, Bisaya, Dusun, Kedayan, Murut and Tutong[49]. Brunei Malay is culturally Malay and embraces Islam, similar to the Malay in West Malaysia, while other indigenous Malays are culturally and religiously diverse[50].

For peer review only - http://bmjopen.bmj.com/site/about/guidelines.xhtml

Social-economic variables (*SES*) included education, employment status and ownership of the house. Education had four groups (primary, secondary, post-secondary and higher degree). Employment status was categorized into employed, unemployed and not in the labour force (i.e., those who are neither in employment nor in unemployment). Being not in the labour force was treated as the reference group. House ownership was a dichotomous variable (1 for owning the house and 0 for not owning the house).

Healthy Lifestyle

Due to limited information, a healthy lifestyle (*HLS*) was measured with a survey question asking about the frequency of doing physical activities: "How often do you play sports or exercise per week?" It had four responses: 1=never, 2= 1 to 2 times, 3=3 to 5 times, and 4= every day. Never playing sport or exercise was the reference category.

Statistical Methods

Chi-square statistics was initially performed through a series of cross-tabulations between self-rated health status and all selected variables. Logistic regression model[51] was used for assessing the association between perceived environmental quality and SRH. Below is the logistics regression model used in this paper.

$$\ln\left(\frac{p}{1-p}\right) = \beta_0 + \beta_j PEQ_j + \beta_k DEM_k + \beta_l SES_l + \beta_m HLS_m + \varepsilon$$

where p refers to probability of good self-rate health (SRH)

PEQ refers to perceived environmental quality with j indicators

DEM refers to demographic control variable, with k indicators

For peer review only - http://bmjopen.bmj.com/site/about/guidelines.xhtml

SES refers to socio-economic variables with *i* indicators

HLS refers to a healthy lifestyle with only 1 indicator

Following the previous study[17], the model was applied in few stages to examine each group of variables at each stage. This paper has four stages to observe the possible changes played by perceived environmental quality, demographic variables, socio-economic variables and lifestyle variables on SRH. Model 1 assessed the association between SRH and *PEQ* without control variables. Model 2 enriched Model 1 by adding demographic variables, Model 3 further added *SES,* while Model 4 added *HLS* as controls. The p=0.05 was selected to determine the significance of the variables. Odds Ratio (OR) and its 95% Confident Intervals (CI) were provided to examine the significance of the relationship.

RESULTS

Sample description

An overview of the characteristics of the respondents based on demographic, socioeconomic variables and environmental quality is presented in Table 1. More than half were female (52.3%) and mature ages between 30-59 years old (51.0%). The majority were married (55.6%), with a significant percentage of single (37.7%). The rest was ever married, either widowed or divorced (6.7%). Brunei Malay accounted for the largest ethnic group (63.3%). More than half attended post-secondary and above (58.5%). About 65% were employed. Less than half did not own the houses they lived in. Regarding a healthy lifestyle, only one fifth never participated in physical exercise, and many of them had their physical activities once or twice per week.

For peer review only - http://bmjopen.bmj.com/site/about/guidelines.xhtml

Table 1 about here

Perceived Environmental Quality

The analysis of the survey data shows a good perception of air quality in the area the respondents reside. The descriptive statistics show that 80.2% of respondents perceived that they reside in fairly good air quality areas. Only 12.5% of respondents perceived they live in an area with very good air quality. In other words, only a smaller percentage of respondents (7.3%) perceived they live in air-polluted areas. They expressed the need to improve the air quality, and half of them were committed to it (Table 2). About 77% did not have problems with sources of water supply in their daily life. The majority (73.7%) did not perceive the existence of other pollutions such as noise, olfactory and others (Table 2). However, a lower percentage, more than half of them (53.8%), perceived that the condition of marine resources in the district was not good. Social environmental quality was reported frequently by 71.9% of residents considering their areas have low crime.

Table 2 about here

Differential of SRH: Chi-square analysis

Overall, the majority (72.7%) perceived good self-rated health (SRH). The Chi-square analysis presented in Table 2 shows that the prevalence of good SRH significantly differed by perceived air quality (X^2=12.9, p=0.002), marine resources (X^2=10.3, p=0.001) and water supply (X^2=5.4, p=0.026). The prevalence of good SRH for respondents living in perceived very clean air areas was higher (77.4%) than those

For peer review only - http://bmjopen.bmj.com/site/about/guidelines.xhtml

living in fairly clean air areas (73.9%) and much higher than those living in polluted or not clean air areas (52.0%). Perceived air quality was thus positively associated with good SRH. However, the prevalence of good SRH was not significantly different between respondents perceiving very importantly to improve air quality and those perceiving not importantly to do so (Table 2). In other words, commitment to improving air quality was not associated with good SRH, though perceived air quality was positively related to good SRH. This indicates the existence of a knowledge-attitude gap.

The prevalence of good SRH was higher among those living in areas perceived to have good marine resources than those living in areas not having good marine resources (78.8% and 67.4%, respectively, $p<0.001$). The prevalence of good SRH was different between respondents having exposure to noise, olfactory and other pollutants and those not being exposed to these pollutants (74.0% and 68.9%, respectively), although the Chi-square result shows no significant difference ($X^2=1.4$, $p=0.230$).

Prevalence of good SRH was higher among those living in a house without any problems with water supply (74.9%) than those having problems with water supply (65.4%, $p<0.005$). Social environment condition, measured with the perception of safety from crime, was not associated with good SRH.

Age mattered for health status. Table 1 shows that the prevalence of good SRH was lower for the older ages than the younger ages. Men and women perceived almost equally good SRH (72.9% and 72.4%, respectively, $X^2=0.002$, $p=0.964$). There was also no ethnic differential in the prevalence of good SRH ($X^2=3.3$, $p=0.349$).

For peer review only - http://bmjopen.bmj.com/site/about/guidelines.xhtml

Among socio-economic variables, employment status is the only variable which significantly differentiated good SRH (X^2=12.6, *p*=0*p*=0.002). The prevalence of good SRH for either employed (76.4%) or unemployed (77.3%) was higher than for those who were not in the labour force (63.0%). Marital status, education and home ownership were not significantly associated with SRH.

Physical exercise mattered more (X^2=23.0, *p*=0.000). Prevalence of good SRH was much higher (88.3%) among respondents exercising regularly between 3 and 5 times per week than those who never did any physical exercise (63.4%).

Logistic regression of self-rated health

There are four logistic regression results presented in Tables 3 and 4. Model 1 examines the association between perception of environmental quality and SRH without controlling for other sets of variables. This model shows that respondents with the perception of living in fairly clean air areas (OR=2.42, 95% CI 1.30 to 4.49, *p*=0.005) or very clean air areas (OR=2.40, 95% CI 1.07 to 5.38, *p*=0.034) were more likely to state in good SRH than those living in the areas with air pollution. This pattern was slightly different from the Chi-square result, with respondents in very clean air areas having the highest prevalence of good SRH. While our further examination (not shown in the table) indicates that the difference between these two groups was insignificant, the association between perception of air quality and good SRH remains significant.

Table 3 about here

For peer review only - http://bmjopen.bmj.com/site/about/guidelines.xhtml

Respondent's opinion and commitment to improving air quality were not associated with SRH (Table 3). Respondents living in areas with perceived good marine resources were more likely to report good SRH than those living in not good condition marine resources (OR=1.69, 95% CI 1.18 to 2.44, *p*=0.005).

However, good health was not significantly associated with respondents' perception of other environmental qualities (water supply; and pollution of noise, olfactory and others). Model 1 changes the significance level of the association between water supply and good SRH into insignificant from the Chi-square result. This indicates that the presence of perception of air quality and marine had a more salient influence on the association.

Neither was the social environmental condition measured by the perception of crime associated with SRH. These findings on the association between perceived environmental quality on good SRH remain the same after controlling for demographic variables (Model 2).

It is worth briefly discussing the findings on demographic variables. Among the selected demographic variables, age was negatively associated with self-rated health (Model 2). The odds of stating good SRH decreased with age (OR=0.98, 95% CI 0.96 to 0.99, *p*=0.001). This is in contrast to the relationship between gender and good SRH with OR=1 and *p*=0.913. In other words, men and women had the same likelihood of reporting good SRH. There was also no difference in the likelihood of reporting good SRH among marital status and ethnic groups. This deserves further study.

For peer review only - http://bmjopen.bmj.com/site/about/guidelines.xhtml

Adding socio-economic variables, in model 3 (Table 4), all associations between perceived environmental quality and SRH remain the same, with a slight change in the magnitude of the perceived air quality and marine resources. The adjusted odds ratios of reporting good SRH were lower in fairly clean air areas (OR=2.35, 95% CI 1.25 to 4.44, *p*=0.008) and higher in very clean air areas (OR=2.44, 95% CI 1.06 to 5.63, *p*=0.036) than the odds ratios presented in unadjusted model (model 1). In other words, model 3 shows a positive relationship between perceived air quality and good SRH, which was similar to the Chi-square result.

Table 4 about here

The association between perceived good marine resources quality and SRH became stronger (OR=1.77, 95% CI 1.20 to 2.60, *p*=0.004 in model 3 compared to OR=1.69 in model 1). In other words, controlling for socio-economic variables, the association between perceived environmental quality (especially air quality and marine resources) and SRH became stronger. Socio-economic variables thus strengthened the relationship.

Among socio-economic variables, employment status was the only variable significantly associated with good SRH. Our data shows that a large proportion (28.5%) of the respondents were not in the labour market. If bad health is one of the reasons for not joining the labour market, it is not surprising that employed respondents were more likely to report good SRH than non-labour force (OR=1.63, 95% CI 1.05 to 2.53, *p*=0.029). This likelihood was not different between unemployed and non-labour force. In addition, the likelihood of reporting good SRH was not significant for educational attainment and home ownership.

For peer review only - http://bmjopen.bmj.com/site/about/guidelines.xhtml

Finally, model 4 shows that a healthy lifestyle measured with the frequency of doing physical exercise played the most prominent association with SRH. Doing physical exercise three to five times per week was beneficial for good health (OR=3.89, 95% CI 1.96 to 7.71, *p*=0.000, the highest odd ratio). However, the health impact of doing the physical exercise once or twice, or even everyday weekly, was not significantly different from those never doing any exercise. This may indicate that too much exercise has more detrimental effects than benefits on health. However, there is no further information related to physical exercise, such as the duration of exercise, which deserves further research.

Interestingly, the inclusion of physical exercise into the model made the association between perceived air quality and SRH insignificant (*p*=0.059). In addition, the odds ratio for respondents perceiving fairly clean air became smaller (OR=2.20, Model 4), declining from 2.35 (Model 3). The association between age and SRH also became insignificant. In other words, physical exercise played a mediating role in the association between perceived environmental quality and good SRH. The inclusion of physical activity strengthened the association between employment status and good SRH as the OR for employed in Model 4 (OR=1.72, 95% CI 1.09 to 2.71, *p*=0.021) was higher than in Model 3.

DISCUSSION

Environmental variables

This study contributes to the literature by empirically investigating the relationship between perceived environmental quality and SRH for Brunei, a Southeast Asian

For peer review only - http://bmjopen.bmj.com/site/about/guidelines.xhtml

country. Our findings suggest the importance of addressing natural environmental qualities across different dimensions to gauge their differential relationships with SRH. Specifically, the study found that residents who perceived clean air and good marine resources in their surrounding areas were more likely to report good SRH than residents who perceived polluted air and polluted marine resources. Our findings align with a previous study that marine quality had a positive relationship with health, which was measured as an objective health[52]. Marine and coastal areas have been extensively polluted with various forms of plastics such as water bottles, plastic bags, children's toys, wrappers, medical waste and many others. Plastics affect marine resources through the release of microplastics as harmful pollutant[52]. Polluted marine may pollute the fish and other consumable sea commodities such as seaweed, shellfish and others. This may have further implications for physical health as many people consume the edible marine resources collected and caught in the sea.

It is therefore important to pay attention to the health impact of marine resources pollution as the possibility of both onshore and offshore oil spills occurring in oil production areas such as the Belait district is very high. The health effect of oil spills could last long, and residents' exposure to hydrocarbon had detrimental effects on foetal development and infant mortality, as shown in the case of Nigeria, an oil-producing country[53]. However, land-based activities such as mining, agriculture, forestry, industrial activities, household consumption, river-based transportation, and chemical industries can also be a major source of marine pollution[54].

Furthermore, residents who perceived living in clean air areas were more likely to report good health. Our findings are consistent with earlier studies[32,33,55]. Poor air

For peer review only

quality could cause respiratory infection[35,56]. Polluted air was also associated with cardiovascular disease, lung cancer, and lung function. As part of the Southeast Asian region, Brunei is prone to forest fires, especially during the dry season[57,58]. Seria, one of the selected sub-district area, was the hotspot area for forest fires[57]. Bushfires have also often become a public health concern in other countries such as Australia[59], Canada[60] and the US[61]. In addition, exposure to transboundary haze from surrounding ASEAN countries was occasionally experienced in Brunei[34].

Both indoor and outdoor air pollution have been a major concern for global public health[56]. Open burning of household rubbish is another source of air pollution. Air pollution, measured objectively by particulate molecule (PM_{10}) exceeding the level for good quality of air in Belait, had the longest recorded days among districts[62]. The lessons from various studies around the Ecuadorian Amazon regions were that crude-oil production activities contributed negative health impacts to communities in the form of various cancers such as rectum, skin, kidney, cervix, stomach, skin, leukaemia, soft tissue, and cervix[63]. Therefore, policy-making needs to consider the surrounding environment to improve people's health.

At the same time, our findings also indicate that commitment to improve air quality was not important in good SRH, though perceived air quality was positively related to good SRH. Perhaps, the respondents believed that the government could do better in preserving the air quality.

Noise pollution among urban residents of China was positively associated with poor SRH[26,32]. However, in the Brunei case, our finding reveals that perceived olfactory (odour) and noise pollutant was not significantly related to health, although

For peer review only - http://bmjopen.bmj.com/site/about/guidelines.xhtml

26.3% of the selected sample perceived to be exposed to olfactory and noise pollutants. It should be noted here that the questionnaire recorded information on "olfactory pollutant" and "noise pollutant" in one group.

A previous study found that the impact of noise on health was mostly from road traffic[64]. Noise from road traffic may result in nervousness and disturb sleep quality. Nevertheless, as road traffic is relatively low in Brunei and Belait in particular, compared to many other countries, noise pollutants may not have any impact on health in our study. Moreover, literature review[65] revealed that there was limited empirical evidence on the association between olfactory pollutants and health. As such, our finding may indicate that olfactory and noise pollution might be a nuisance but did not necessarily affect health.

Non-environmental variables

Another important finding is that a healthy lifestyle such as frequent physical exercise can compensate for the health impact of perceived environmental quality. Physical activity mediated the relationship between perceived air quality and SRH. Our findings show that if residents actively and regularly practiced physical exercise, the difference in the prevalence of good SRH became insignificant between residents living in perceived clean air and polluted air. In other words, regular physical activity can mitigate the adverse environmental consequences on health. Our findings reveal that regular exercise was positively associated with good SRH, confirmed by earlier studies[22,41,47].

For peer review only - http://bmjopen.bmj.com/site/about/guidelines.xhtml

Previous studies showed that the association between age and health was non-conclusive. Age was not significantly associated with SRH[26]. On the other hand, in our study, age was negatively associated with good SRH when the model was not controlled with physical activity. This means that as people became older, the prevalence of good health declined, supporting earlier studies[24,32,41,46].

However, our finding further suggests a mediating effect of physical activity on the relationship between age and SRH, which turned this relationship insignificant. In other words, for the same level of physical activity, age did no longer differentiate SRH. The result indicates that the older people who exercised regularly may have an insignificant difference in good health compared to younger people. In other words, a physical activity plays a very important role in SRH for all ages.

Another important variable associated with good SRH was employment. Previous studies indicated that work-health relationships can be simultaneous. The relationship can be a result of bias in which only healthy people are willing to work, and unhealthy ones are leaving their jobs. Poor SRH can thus be associated with being the unemployed or increasing risk of job loss[66]. On the other hand, the relationship between employment and health had mixed results; being employed can be associated with good SRH, bad SRH or no relationship[66]. Being employed in poorly paid jobs or stressful jobs can be associated with poor SRH. However, our study found a positive association -- being employed was more likely to be good SRH (OR=1.72, 95% CI 1.09 to 2.71, p=0.021) than being out of the labour force or unemployed.

Conclusion

For peer review only - http://bmjopen.bmj.com/site/about/guidelines.xhtml

Our study concludes that perception of marine resources and air quality mattered to the residents' self-rated health status. Therefore, special attention must be spent to improving air quality and marine resources. Moreover, our findings also show that physical exercise and employment play important roles in health. There is a strong need for policy intervention to mitigate the negative health impact of environmental quality through promoting physical exercise and employment. Further studies should explore more comprehensive measurements of environmental quality, healthy lifestyle and employment variables in assessing health and environmental quality.

Acknowledgements

The authors would like to thank Brunei Shell Petroleum (BSP) Sendirian Berhad for providing the funding to conduct this study. The authors would also like to thank Professor Gour Dasvarma for valuable input and the field research team who worked hard to complete the data collection.

Author Contributions. ENA designed the concept, analysed data, chose the method, and wrote the manuscript. C-YH supervised project administration and data collection, wrote, reviewed and edited the final draft. LS coordinated the data collection, reviewed and edited the final draft. AT facilitated funding acquisition, analysed data, reviewed the methods and edited the final draft. All authors contributed to read, edit and approve the final draft of the manuscript.

A Competing interests statement. None declared.

Funding statement. The work was supported by a research grant sponsored by the Brunei Shell Petroleum (BSP) Sendirian Berhad with the reference no.: BSP/SI2018.011/CEA4. The funder had no role in the study design, data collection and analysis, decision to publish, or preparation of the manuscript. The authors independently analysed the anonymous data.

Data statement. No data are available to public due to data agreement.

For peer review only - http://bmjopen.bmj.com/site/about/guidelines.xhtml

Patient consent for publication Not required.

Informed consent. Respondents did give informed consent.

Word count: 4699 words

References

1. Ministry of Finance and Economy. *Voluntary National Review Report of Brunei Darussalam*. Ministry of Finance and Economy, Government of Brunei Darussalam; 2020.

2. Buddelmeyer H, Cai L. Interrelated Dynamics of Health and Poverty in Australia. *SSRN Electron J*. 2021;(4602). doi:10.2139/ssrn.1519238

3. Clarke P, Erreygers G. Defining and measuring health poverty. *Soc Sci Med*. 2020;244(February 2019):112633. doi:10.1016/j.socscimed.2019.112633

4. Ananta A, Moeis AIA, Widianto HT, Yulianto H, Arifin EN. Pension and Active Ageing: Lessons Learned from Civil Servants in Indonesia. *Soc Sci*. 2021;10(11):436. doi:10.3390/socsci10110436

5. Dang HA, Lanjouw P, Vrijburg E. Poverty in India in the face of Covid-19: Diagnosis and prospects*. *Rev Dev Econ*. 2021;25(4):1816-1837. doi:10.1111/rode.12833

6. Olivia S, Gibson J, Nasrudin R. Indonesia in the Time of Covid-19. *Bull Indones Econ Stud*. 2020;56(2):143-174. doi:10.1080/00074918.2020.1798581

7. Whitehead M, Taylor-Robinson D, Barr B. Poverty, health, and covid-19. *BMJ*. 2021;372:1-2. doi:10.1136/bmj.n376

8. Craig BA, Morton DP, Morey PJ, et al. The association between self-rated

For peer review only - http://bmjopen.bmj.com/site/about/guidelines.xhtml

health and social environments, health behaviors and health outcomes: A structural equation analysis. *BMC Public Health*. 2018;18(1):1-10. doi:10.1186/s12889-018-5323-y

9. Hoeymans N, Feskens EJM, Kromhout D, Van Den Bos GAM. The contribution of chronic conditions and disabilities to poor self- rated health in elderly men. *Journals Gerontol - Ser A Biol Sci Med Sci*. 1999;54(10):501-506. doi:10.1093/gerona/54.10.M501

10. Lee HW, Song M, Yang JJ, Kang D. Determinants of poor self-rated health in Korean adults with diabetes. *J Prev Med Public Heal*. 2015;48(6):287-300. doi:10.3961/jpmph.15.048

11. Li Y, Nima Q, Yu B, et al. Determinants of self-rated health among an older Tibetan population in a Chinese plateau area: analysis based on the conceptual framework for determinants of health. *BMC Public Health*. 2021;21(1):1-11. doi:10.1186/s12889-021-10359-x

12. Kim JH, Kim KR, Cho KH, Yoo KB, Kwon JA, Park EC. The association between sleep duration and self-rated health in the Korean general population. *J Clin Sleep Med*. 2013;9(10):1057-1064. doi:10.5664/jcsm.3082

13. Pikhart H. *Social and Psychosocial Determinants of Self-Rated Health in Central and Eastern Europe*.; 2002. doi:10.1007/978-1-4615-0839-7

14. Wuorela M, Lavonius S, Salminen M, Vahlberg T, Viitanen M, Viikari L. Self-rated health and objective health status as predictors of all-cause mortality among older people: A prospective study with a 5-, 10-, and 27-year follow-up. *BMC Geriatr*. 2020;20(1):1-7. doi:10.1186/s12877-020-01516-9

For peer review only

15. Krueger PM, Dovel K, Denney JT. Democracy and self-rated health across 67 countries: A multilevel analysis. *Soc Sci Med*. 2015;143:137-144. doi:10.1016/j.socscimed.2015.08.047

16. Chen H, Liu Y, Zhu Z, Li Z. Does where you live matter to your health? Investigating factors that influence the self-rated health of urban and rural Chinese residents: Evidence drawn from Chinese General Social Survey data. *Health Qual Life Outcomes*. 2017;15(1):1-11. doi:10.1186/s12955-017-0658-0

17. Park JH, Lee KS. Self-rated health and its determinants in Japan and South Korea. *Public Health*. 2013;127(9):834-843. doi:10.1016/j.puhe.2012.12.012

18. Baidin V, Gerry CJ, Kaneva M. How Self-Rated is Self-Rated Health? Exploring the Role of Individual and Institutional Factors in Reporting Heterogeneity in Russia. *Soc Indic Res*. 2021;155(2). doi:10.1007/s11205-020-02604-4

19. Cullati S, Mukhopadhyay S, Sieber S, Chakraborty A, Burton-Jeangros C. Is the single self-rated health item reliable in India? A construct validity study. *BMJ Glob Heal*. 2018;3(6):1-12. doi:10.1136/bmjgh-2018-000856

20. Haddock CK, Poston WSC, Pyle SA, et al. The validity of self-rated health as a measure of health status among young military personnel: Evidence from a cross-sectional survey. *Health Qual Life Outcomes*. 2006;4:1-9. doi:10.1186/1477-7525-4-57

21. Idler EL, Benyamini Y. Self-Rated Health and Mortality : A Review of Twenty-Seven Community Studies Author (s): Ellen L . Idler and Yael Benyamini Source : Journal of Health and Social Behavior , Vol . 38 , No . 1 (Mar ., 1997), pp . 21-37 Published by : American Sociologic. *J Health Soc Behav*. 1997;38(1):21-

For peer review only - http://bmjopen.bmj.com/site/about/guidelines.xhtml

37.

22. Jradi H, Alharbi Z, Mohammad Y. Self-rated health among Saudi women: Association with morbidity, lifestyle, and psychosocial factors. *J Epidemiol Glob Health*. 2018;8(3-4):183-188. doi:10.2991/j.jegh.2018.04.100

23. Vie TL, Hufthammer KO, Meland E, Breidablik HJ. Self-rated health (SRH) in young people and causes of death and mortality in young adulthood. A prospective registry-based Norwegian HUNT-study. *SSM - Popul Heal*. 2019;7(January):100364. doi:10.1016/j.ssmph.2019.100364

24. Cummins S, Stafford M, Macintyre S, Marmot M, Ellaway A. Neighbourhood environment and its association with self rated health: Evidence from Scotland and England. *J Epidemiol Community Health*. 2005;59(3):207-213. doi:10.1136/jech.2003.016147

25. Hale L, Hill TD, Friedman E, et al. Perceived neighborhood quality, sleep quality, and health status: Evidence from the Survey of the Health of Wisconsin. *Soc Sci Med*. 2013;79(1):16-22. doi:10.1016/j.socscimed.2012.07.021

26. Ou JY, Peters JL, Levy JI, Bongiovanni R, Rossini A, Scammell MK. Self-rated health and its association with perceived environmental hazards, the social environment, and cultural stressors in an environmental justice population. *BMC Public Health*. 2018;18(1):1-10. doi:10.1186/s12889-018-5797-7

27. Patel K V., Eschbach K, Rudkin LL, Peek MK, Markides KS. Neighborhood context and self-rated health in older Mexican Americans. *Ann Epidemiol*. 2003;13(9):620-628. doi:10.1016/S1047-2797(03)00060-7

28. Poortinga W, Dunstan FD, Fone DL. Perceptions of the neighbourhood

For peer review only

environment and self rated health: A multilevel analysis of the Caerphilly Health and Social Needs Study. *BMC Public Health*. 2007;7:1-10. doi:10.1186/1471-2458-7-285

29. Roh S, Jang Y, Chiriboga DA, Kwag KH, Cho S, Bernstein K. Perceived neighborhood environment affecting physical and mental health: A study with Korean American older adults in New York City. *J Immigr Minor Heal*. 2011;13(6):1005-1012. doi:10.1007/s10903-011-9492-3

30. Saikia U, Dodd MM, Chalmers J, Dasvarma G, Schech S. COVID-19, individual wellbeing and multidimensional poverty in the state of South Australia. *PLoS One*. 2021;16(6 June):1-17. doi:10.1371/journal.pone.0252898

31. Perera B, Østbye T, Jayawardana C. Neighborhood environment and self-rated health among adults in southern Sri Lanka. *Int J Environ Res Public Health*. 2009;6(8):2102-2112. doi:10.3390/ijerph6082102

32. Yang T. Association between perceived environmental pollution and health among urban and rural residents-a Chinese national study. *BMC Public Health*. 2020;20(1):1-10. doi:10.1186/s12889-020-8204-0

33. Liu J, Luo Y, Haller W, Mey B Vander, Granberg E. Neighborhood environments and self-rated health in Mainland China, Japan and South Korea. *PLoS One*. 2018;13(9):1-17. doi:10.1371/journal.pone.0204910

34. Dotse SQ, Dagar L, Petra MI, De Silva LC. Influence of Southeast Asian Haze episodes on high PM10 concentrations across Brunei Darussalam. *Environ Pollut*. 2016;219:337-352. doi:10.1016/j.envpol.2016.10.059

35. Luong LMT, Sly PD, Thai PK, Phung D. Impact of ambient air pollution and

For peer review only - http://bmjopen.bmj.com/site/about/guidelines.xhtml

wheeze-associated disorders in children in Southeast Asia: A systematic review and meta-analysis. *Rev Environ Health*. 2019;34(2):125-139. doi:10.1515/reveh-2018-0079

36. Bowe B, Xie Y, Li T, Yan Y, Xian H, Al-Aly Z. Estimates of the 2016 global burden of kidney disease attributable to ambient fine particulate matter air pollution. *BMJ Open*. 2019;9(5). doi:10.1136/bmjopen-2018-022450

37. United Nations. *Human Development Report 2019*.; 2019.

38. von Elm E, Altman DG, Egger M, Pocock SJ, Gøtzsche PC, Vandenbroucke JP. The strengthening the reporting of observational studies in epidemiology (STROBE) statement: Guidelines for reporting observational studies. *Int J Surg*. 2014;12(12):1495-1499. doi:10.1016/j.ijsu.2014.07.013

39. Department of Economic Planning and Statistics BD. Report of the Mid-Year Population Estimates 2020. Published online 2021.

40. DEPD. *Population and Housing Census Update Final Report 2016*. Department of Economic Planning and Development (DEPD), Ministry of Finance and Economy; 2018. https://deps.mofe.gov.bn/DEPD Documents Library/DOS/KBPP/finalreport2016/KBPP_2016.pdf

41. Rütten A, Abel T, Kannas L, et al. Self reported physical activity, public health, and perceived environment: Results from a comparative European study. *J Epidemiol Community Health*. 2001;55(2):139-146. doi:10.1136/jech.55.2.139

42. Harschel AK, Schaap LA, Iwarsson S, Horstmann V, Tomsone S. Self-rated health among very old people in European countries: An explorative study in Latvia and Sweden. *Gerontol Geriatr Med*. 2015;(January-December).

For peer review only

doi:10.1177/2333721415598432

43. Government of Assam, UNDP NA. Assam Human Development Report 2014: Managing Diversities, Achiveing Human Development. *Gov Assam*. Published online 2014:1-306.

44. Vie TL, Hufthammer KO, Holmen TL, Meland E, Breidablik HJ. Is self-rated health in adolescence a predictor of prescribed medication in adulthood? Findings from the Nord Trøndelag Health Study and the Norwegian Prescription Database. *SSM - Popul Heal*. 2018;4(November 2017):144-152. doi:10.1016/j.ssmph.2017.11.010

45. UNDP. *National Human Development Report 2018 - Timor-Leste.*; 2018. doi:10.18356/28cccb7e-en

46. Choi JW, Kim J, Han E, Kim TH. Association of employment status and rated health among income with self- - waged workers with disabilities in South Korea : population- - based panel study. Published online 2019:1-6. doi:10.1136/bmjopen-2019-032174

47. Stronegger WJ, Titze S, Oja P. Perceived characteristics of the neighborhood and its association with physical activity behavior and self-rated health. *Heal Place*. 2010;16(4):736-743. doi:10.1016/j.healthplace.2010.03.005

48. Wen M, Hawkley LC, Cacioppo JT. Objective and perceived neighborhood environment, individual SES and psychosocial factors, and self-rated health: An analysis of older adults in Cook County, Illinois. *Soc Sci Med*. 2006;63(10):2575-2590. doi:10.1016/j.socscimed.2006.06.025

49. Department of Economic Planning and Statistics. *Brunei Darussalam Statistical*

For peer review only

Yearbook 2020.; 2021. https://deps.mofe.gov.bn/DEPD Documents Library/DOS/BDSYB/BDSYB_2020/BDSYB 2020 Final.pdf

50. Loo SP. Ethnicity and Educational Policies in Malaysia and Brunei Darussalam. 2009;6(2):180-192. https://www.academia.edu/710806/Ethnicity_and_educational_policies_in_Malaysia_and_Brunei_Darussalam

51. Hosmer DW, Lemeshow S, Sturdivant RX. *Applied Logistic Regression: Third Edition.*; 2013. doi:10.1002/9781118548387

52. Krishna R, Mohan M, Mallika P, Costa D. Microplastics in ballast water as an emerging source and vector for harmful chemicals , antibiotics , metals , bacterial pathogens and HAB species : A potential risk to the marine environment and human health. *Mar Pollut Bull*. 2019;149(July):110525. doi:10.1016/j.marpolbul.2019.110525

53. Bruederle A, Hodler R. Effect of oil spills on infant mortality in Nigeria. *Proc Natl Acad Sci U S A*. 2019;116(12):5467-5471. doi:10.1073/pnas.1818303116

54. Willis KA, Serra-Gonçalves C, Richardson K, et al. Cleaner seas: reducing marine pollution. *Rev Fish Biol Fish*. 2021;8. doi:10.1007/s11160-021-09674-8

55. Liu P, Wang J, Wang X, Nie W, Zhen F. Measuring the association of self-perceived physical and social neighborhood environment with health of Chinese rural residents. *Int J Environ Res Public Health*. 2021;18(16):1-12. doi:10.3390/ijerph18168380

56. Khaltaev N, Axelrod S. Chronic respiratory diseases global mortality trends, treatment guidelines, life style modifications, and air pollution: Preliminary

For peer review only

For peer review only - http://bmjopen.bmj.com/site/about/guidelines.xhtml

analysis. *J Thorac Dis*. 2019;11(6):2643-2655. doi:10.21037/jtd.2019.06.08

57. Radojevic M, Hassan H. Air quality in Brunei Darussalam during the 1998 haze episode. *Atmos Environ*. 1999;33(22):3651-3658. doi:10.1016/S1352-2310(99)00118-1

58. Shahriar S, El-Said M. M. Z, Safwanah N. M. S, Kho J. H, Lee NNDHMF, Hasim NHH. Risk assessment for forest fire in Brunei Darussalam. *MATEC Web Conf*. 2019;258:05033. doi:10.1051/matecconf/201925805033

59. Borchers Arriagada N, Palmer AJ, Bowman DMJS, Morgan GG, Jalaludin BB, Johnston FH. Unprecedented smoke-related health burden associated with the 2019–20 bushfires in eastern Australia. *Med J Aust*. 2020;213(6):282-283. doi:10.5694/mja2.50545

60. Matz CJ, Egyed M, Xi G, et al. Health impact analysis of PM2.5 from wildfire smoke in Canada (2013–2015, 2017–2018). *Sci Total Environ*. 2020;725:138506. doi:10.1016/j.scitotenv.2020.138506

61. Fann N, Alman B, Broome RA, et al. The health impacts and economic value of wildland fire episodes in the U.S.: 2008–2012. *Sci Total Environ*. 2018;610-611:802-809. doi:10.1016/j.scitotenv.2017.08.024

62. Aziz EN, Kasem A, Haji Suhaili WS, Zhao P. Convolution recurrent neural network for daily forecast of PM10 concentrations in Brunei Darussalam. *Chem Eng Trans*. 2021;83(February):355-360. doi:10.3303/CET2183060

63. Coronel Vargas G, Au WW, Izzotti A. Public health issues from crude-oil production in the Ecuadorian Amazon territories. *Sci Total Environ*. 2020;719:134647. doi:10.1016/j.scitotenv.2019.134647

For peer review only

64. Geravandi S, Takdastan A, Zallaghi E, et al. Noise Pollution and Health Effects. *Jundishapur J Heal Sci*. 2015;7(1):1-15. doi:10.5812/jjhs.25357

65. Guadalupe-Fernandez V, De Sario M, Vecchi S, et al. Industrial odour pollution and human health: a systematic review and meta-analysis. *Environ Heal A Glob Access Sci Source*. 2021;20(1):1-21. doi:10.1186/s12940-021-00774-3

66. Antonisse L, Garfield R. The Relationship Between Work and Health: Findings from a Literature Review. *Issue Br*. 2018;(August). https://www.kff.org/medicaid/issue-brief/the-relationship-between-work-and-health-findings-from-a-literature-review/

For peer review only

For peer review only - http://bmjopen.bmj.com/site/about/guidelines.xhtml

Table 1. Respondents Characteristics and Comparison in Good SRH

Variable		Frequency	Percent	Good SRH	X^2 *value (p)*
	Total	673	100.0	-	
Self-rated health	Not good	184	27.3	-	
	Good health	489	72.7	-	
Sex	Female	352	52.3	72.4	0.002 (0.964)
	Male	321	47.7	72.9	
Age	18-29	241	35.8	77.2	7.785 (0.020)
	30-59	343	51.0	72.3	
	60-85	89	13.2	61.8	
Marital status	Married	374	55.6	71.9	2.453 (0.293)
	Ever married	45	6.7	64.4	
	Single	254	37.7	75.2	
Ethnic groups	Brunei Malay	426	63.3	74.9	3.292 (0.349)
	Indigenous Malay	116	17.2	70.7	
	Chinese	75	11.1	66.7	
	Others	56	8.3	67.9	
Education	Primary and below	37	5.5	54.1	6.926 (0.074)
	Secondary	242	36.0	74.0	
	Post-secondary & diploma	221	32.8	74.2	
	High degree	173	25.7	72.8	
House ownership	Own house	304	45.2	70.1	1.878 (0.171)
	Not own	369	54.8	74.8	
Employment status	Employed	437	64.9	76.4	12.578 (0.002)
	Unemployed	44	6.5	77.3	
	Not labour force	192	28.5	63.0	
Physical exercise	Never	142	21.1	63.4	22.956 (0.000)
	1-2 times	362	53.8	70.4	
	3-5 times	128	19.0	88.3	
	Everyday	41	6.1	75.6	

Source: Authors' calculation based on data collected for this study.

For peer review only - http://bmjopen.bmj.com/site/about/guidelines.xhtml

Table 2. Differential of Good SRH by Perceived Environmental Quality

		Frequency	Percent	Good SRH	X^2 *value (p)*
Air quality	Very clean	84	12.5	77.4	
	Fairly clean	540	80.2	73.9	12.903 (0.002)
	Not clean	49	7.3	51.0	
Water problem	No problem	517	76.8	74.9	5.410 (0.026)
	Water supply problem	156	23.2	65.4	
Marine	Not good	362	53.8	67.4	10.330 (0.001)
	Good	311	46.2	78.8	
Other pollutions	Not exposed	496	73.7	74.0	1.440 (0.230)
	Exposed to noise, odour, pollution problems	177	26.3	68.9	
Crime	Low crime	484	71.9	73.3	0.296 (0.586)
	Medium & high	189	28.1	70.9	
Air quality improvement	Not very/not important	169	25.1	71.6	0.067 (0.796)
	Important to improve air quality	504	74.9	73.0	
Commitment	Less committed	313	46.5	72.2	0.026 (0.873)
	Committed	360	53.5	73.1	

Source: Authors' calculation based on data collected for this study.

review only

Table 3. Odds Ratios for Good SRH and Perceived Environmental Quality controlled for Demographic Variables

Variables	Model 1			Model 2		
	OR	95% CI	Sig.	OR	95% CI	Sig.
**Perceived environmental**						
Air quality (ref=Not clean)			0.020			0.024
Very clean	2.40	1.07 to 5.38	0.034	2.37	1.04 to 5.41	0.040
Fairly clean	2.42	1.30 to 4.49	0.005	2.39	1.28 to 4.49	0.007
Marine Resources (ref=Not good)						
Good Marine Resources	1.69	1.18 to 2.44	0.005	1.78	1.22 to 2.60	0.003
Other pollutions (ref=Not expose)						
Exposed Noise, odour, & others	1.03	0.68 to 1.55	0.907	1.02	0.68 to 1.56	0.908
Water supply (ref=No problem)						
Problem with water supply	0.71	0.47 to 1.08	0.110	0.74	0.49 to 1.13	0.166
Crime (ref=Low crime)						
Medium and High crime	0.98	0.67 to 1.45	0.933	0.90	0.61 to 1.34	0.610
Air Quality Improvement (ref=Not important)						
Important	0.96	0.64 to 1.45	0.857	0.96	0.63 to 1.45	0.830
Commitment to Air Quality Improvement (ref=Not committed)						
Committed	1.08	0.75 to 1.54	0.687	1.02	0.71 to 1.46	0.934
**Demographic variables**						
Age (in years)	-	-	-	0.98	0.96 to 0.99	0.001
Sex (ref=Female)						
Male	-	-	-	1.02	0.71 to 1.46	0.913
Ethnic group (ref=Brunei Malay)						0.588
Indigenous Malay	-	-	-	0.82	0.51 to 1.31	0.397
Chinese	-	-	-	0.86	0.49 to 1.51	0.597
Others	-	-	-	0.68	0.37 to 1.28	0.231
Marital status (ref=Married)						0.409
Ever married	-	-	-	0.86	0.42 to 1.74	0.669
Single	-	-	-	0.72	0.44 to 1.19	0.201
Constant	1.02		0.967	3.70		0.018

Notes: Ref = reference group

Source: Authors' calculation based on data collected for this study.

For peer review only

Table 4. Odds Ratios for Good SRH and Perceived Environmental Quality controlled for Demographic-Socio-Economic and Healthy Lifestyle Variables

Variables	Model 3			Model 4		
	OR	95% CI	Sig.	OR	95% CI	Sig.
**Perceived environmental**						
Air quality (ref=Not clean)			0.029			0.059
Very clean	2.44	1.06 to 5.63	0.036	2.19	0.93 to 5.12	0.072
Fairly clean	2.35	1.25 to 4.44	0.008	2.20	1.15 to 4.22	0.018
Marine Resources (ref=Not good)						
Good Marine Resources	1.77	1.20 to 2.60	0.004	1.84	1.24 to 2.74	0.002
Other pollutions (ref=Not expose)						
Exposed Noise, odour, & others	1.07	0.70 to 1.63	0.771	0.98	0.63 to 1.51	0.911
Water supply (ref=No problem)						
Problem with water supply	0.72	0.47 to 1.11	0.136	0.71	0.46 to 1.10	0.123
Crime (ref=Low crime)						
Medium and High crime	0.90	0.60 to 1.34	0.595	0.97	0.64 to 1.45	0.866
Air Quality Improvement (ref=Not important)						
Important	0.97	0.64 to 1.47	0.874	0.98	0.64 to 1.50	0.926
Commitment to Air Quality Improvement (ref=Not committed)						
Committed	0.99	0.69 to 1.44	0.975	0.92	0.63 to 1.35	0.681
**Demographic variables**						
Age (in years)	0.98	0.96 to 0.99	0.033	0.98	0.97 to 1.00	0.079
Sex (ref=Female)						
Male	0.98	0.68 to 1.42	0.912	0.86	0.59 to 1.26	0.433
Ethnic group (ref=Brunei Malay)			0.599			0.384
Indigenous Malay	0.78	0.49 to 1.26	0.314	0.78	0.48 to 1.26	0.303
Chinese	0.87	0.49 to 1.54	0.632	0.83	0.46 to 1.48	0.519
Others	0.71	0.37 to 1.34	0.287	0.60	0.31 to 1.16	0.126
Marital status (ref=Married)	-		0.585	-		0.552
Ever married	0.89	0.42 to 1.86	0.747	0.77	0.36 to 1.63	0.487
Single	0.77	0.46 to 1.29	0.320	0.79	0.47 to 1.34	0.384
**Socio-economic variables**						
Education (ref=Primary and below)			0.550			0.435
Secondary	1.35	0.60 to 2.99	0.468	1.27	0.56 to 2.88	0.567
Post-secondary and Diploma	1.04	0.438 to 2.46	0.934	0.93	0.39 to 2.26	0.876
Higher education	0.99	0.411to 2.38	0.981	0.87	0.35 to 2.14	0.755
Employment (ref=Not labour force)			0.091			0.068
Employed	1.63	1.05 to 2.53	0.029	1.72	1.09 to 2.71	0.021
Unemployed	1.53	0.66 to 3.57	0.323	1.63	0.68 to 3.89	0.270
House ownership (ref=Not own)						
Own	1.09	0.73 to 1.63	0.669	1.11	0.60 to 1.36	0.626
**Healthy lifestyle**<br>Physical activities (ref=Never)						0.000
1-2 times	-	-	-	1.06	0.67 to 1.68	0.795
3-5 times	-	-	-	3.89	1.96 to 7.71	0.000
Everyday	-	-	-	1.94	0.83 to 4.50	0.124
Constant	1.76		0.466	1.47		0.633

For peer review only - http://bmjopen.bmj.com/site/about/guidelines.xhtml

Source: Authors' calculation based on data collected for this study.

For peer review only

For peer review only - http://bmjopen.bmj.com/site/about/guidelines.xhtml

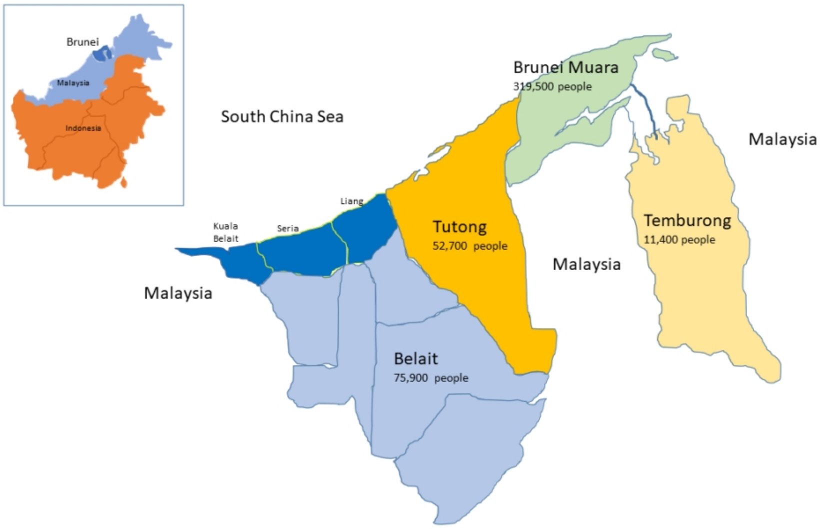

Figure 1. Geographical location of the studied areas

338x190mm (96 x 96 DPI)

Reporting checklist for cross sectional study.

Based on the STROBE cross sectional guidelines.

Instructions to authors

Complete this checklist by entering the page numbers from your manuscript where readers will find each of the items listed below.

Your article may not currently address all the items on the checklist. Please modify your text to include the missing information. If you are certain that an item does not apply, please write "n/a" and provide a short explanation.

Upload your completed checklist as an extra file when you submit to a journal.

In your methods section, say that you used the STROBE cross sectionalreporting guidelines, and cite them as:

von Elm E, Altman DG, Egger M, Pocock SJ, Gotzsche PC, Vandenbroucke JP. The Strengthening the Reporting of Observational Studies in Epidemiology (STROBE) Statement: guidelines for reporting observational studies.

		Reporting Item	Page Number
**Title and abstract**			
Title	#1a	Indicate the study's design with a commonly used term in the title or the abstract	1

For peer review only

Abstract	#1b	Provide in the abstract an informative and balanced summary of what was done and what was found	1-2
**Introduction**			
Background / rationale	#2	Explain the scientific background and rationale for the investigation being reported	3-6
Objectives	#3	State specific objectives, including any prespecified hypotheses	6
**Methods**			
Study design	#4	Present key elements of study design early in the paper	7
Setting	#5	Describe the setting, locations, and relevant dates, including periods of recruitment, exposure, follow-up, and data collection	7
Eligibility criteria	#6a	Give the eligibility criteria, and the sources and methods of selection of participants.	8
	#7	Clearly define all outcomes, exposures, predictors, potential confounders, and effect modifiers. Give diagnostic criteria, if applicable	8-11
Data sources / measurement	#8	For each variable of interest give sources of data and details of methods of assessment (measurement). Describe comparability of assessment methods if there is more than one group. Give information separately for for exposed and unexposed groups if applicable.	8-11

For peer review only

Bias	#9	Describe any efforts to address potential sources of bias	7-8
Study size	#10	Explain how the study size was arrived at	8
Quantitative variables	#11	Explain how quantitative variables were handled in the analyses. If applicable, describe which groupings were chosen, and why	10-11
Statistical methods	#12a	Describe all statistical methods, including those used to control for confounding	10-13
Statistical methods	#12b	Describe any methods used to examine subgroups and interactions	10-13
Statistical methods	#12c	Explain how missing data were addressed	8
Statistical methods	#12d	If applicable, describe analytical methods taking account of sampling strategy	-
Statistical methods	#12e	Describe any sensitivity analyses	-
**Results**			
Participants	#13a	Report numbers of individuals at each stage of study—eg numbers potentially eligible, examined for eligibility, confirmed eligible, included in the study, completing follow-up, and analysed. Give information separately for for exposed and unexposed groups if applicable.	13
Participants	#13b	Give reasons for non-participation at each stage	13

For peer review only

Participants	#13c	Consider use of a flow diagram	
Descriptive data	#14a	Give characteristics of study participants (eg demographic, clinical, social) and information on exposures and potential confounders. Give information separately for exposed and unexposed groups if applicable.	13
Descriptive data	#14b	Indicate number of participants with missing data for each variable of interest	13
Outcome data	#15	Report numbers of outcome events or summary measures. Give information separately for exposed and unexposed groups if applicable.	13
Main results	#16a	Give unadjusted estimates and, if applicable, confounder-adjusted estimates and their precision (eg, 95% confidence interval). Make clear which confounders were adjusted for and why they were included	13-23
Main results	#16b	Report category boundaries when continuous variables were categorized	13-23
Main results	#16c	If relevant, consider translating estimates of relative risk into absolute risk for a meaningful time period	13-23
Other analyses	#17	Report other analyses done—e.g., analyses of subgroups and interactions, and sensitivity analyses	
**Discussion**			
Key results	#18	Summarise key results with reference to study objectives	23-24

For peer review only

Limitations	#19	Discuss limitations of the study, taking into account sources of potential bias or imprecision. Discuss both direction and magnitude of any potential bias.	13-23
Interpretation	#20	Give a cautious overall interpretation considering objectives, limitations, multiplicity of analyses, results from similar studies, and other relevant evidence.	13-23
Generalisability	#21	Discuss the generalisability (external validity) of the study results	23
**Other Information**			
Funding	#22	Give the source of funding and the role of the funders for the present study and, if applicable, for the original study on which the present article is based	24

None The STROBE checklist is distributed under the terms of the Creative Commons Attribution License CC-BY. This checklist can be completed online using https://www.goodreports.org/, a tool made by the EQUATOR Network in collaboration with Penelope.ai

For peer review only

Self-rated Health and Perceived Environmental Quality in Brunei Darussalam:A cross-sectional study

Journal:	*BMJ Open*
Manuscript ID	bmjopen-2022-060799.R2
Article Type:	Original research
Date Submitted by the Author:	01-Jul-2022
Complete List of Authors:	Arifin, Evi Nurvidya; Universiti Brunei Darussalam, Centre for Advanced Research (CARe)<br>Hoon, Chang-Yau; Universiti Brunei Darussalam, Centre for Advanced Research (CARe)<br>Slesman, Ly; Universiti Brunei Darussalam, Centre for Advanced Research (CARe)<br>Tan, Abby; Universiti Brunei Darussalam, Chancellery
**Primary Subject Heading**:	Epidemiology
Secondary Subject Heading:	Health policy, Public health
Keywords:	EPIDEMIOLOGY, PUBLIC HEALTH, PRIMARY CARE

SCHOLARONE™
Manuscripts

I, the Submitting Author has the right to grant and does grant on behalf of all authors of the Work (as defined in the below author licence), an exclusive licence and/or a non-exclusive licence for contributions from authors who are: i) UK Crown employees; ii) where BMJ has agreed a CC-BY licence shall apply, and/or iii) in accordance with the terms applicable for US Federal Government officers or employees acting as part of their official duties; on a worldwide, perpetual, irrevocable, royalty-free basis to BMJ Publishing Group Ltd ("BMJ") its licensees and where the relevant Journal is co-owned by BMJ to the co-owners of the Journal, to publish the Work in this journal and any other BMJ products and to exploit all rights, as set out in our licence.

The Submitting Author accepts and understands that any supply made under these terms is made by BMJ to the Submitting Author unless you are acting as an employee on behalf of your employer or a postgraduate student of an affiliated institution which is paying any applicable article publishing charge ("APC") for Open Access articles. Where the Submitting Author wishes to make the Work available on an Open Access basis (and intends to pay the relevant APC), the terms of reuse of such Open Access shall be governed by a Creative Commons licence – details of these licences and which Creative Commons licence will apply to this Work are set out in our licence referred to above.

Other than as permitted in any relevant BMJ Author's Self Archiving Policies, I confirm this Work has not been accepted for publication elsewhere, is not being considered for publication elsewhere and does not duplicate material already published. I confirm all authors consent to publication of this Work and authorise the granting of this licence.

For peer review only

Self-rated Health and Perceived Environmental Quality in Brunei Darussalam: A cross-sectional study

Evi Nurvidya Arifin*
Centre for Advanced Research (CARe), Universiti Brunei Darussalam,
Jalan Tungku Link, Gadong BE1410, Brunei Darussalam
Email: evi.arifin@ubd.edu.bn

Chang-Yau Hoon
Centre for Advanced Research (CARe), Universiti Brunei Darussalam,
Jalan Tungku Link, Gadong BE1410, Brunei Darussalam
Email: changyau.hoon@ubd.edu.bn

Ly Slesman
Centre for Advanced Research (CARe), Universiti Brunei Darussalam,
Jalan Tungku Link, Gadong BE1410, Brunei Darussalam
Email: slesman.ly@ubd.edu.bn

Abby Tan
Chancellery, Universiti Brunei Darussalam,
Jalan Tungku Link, Gadong BE1410, Brunei Darussalam
Email: abby.tan@ubd.edu.bn

Corresponding author: Evi Nurvidya Arifin, Centre for Advanced Research (CARe), Universiti Brunei Darussalam, Jalan Tungku Link, Gadong BE1410, Brunei Darussalam. Email address: evi.arifin@ubd.edu.bn. Phone: +673 246 0922/3 Ext 2741.

For peer review only

ABSTRACT

Objectives: This paper examines the relationship between individuals' perceptions of environmental quality and self-rated health (SRH) after controlling for dimensions of socio-economic-demographic and healthy lifestyle variables.
Design: A cross-sectional survey.
Setting: The survey was conducted in Belait, an oil- and gas-rich district, Brunei Darussalam, from 17 October to 11 November 2019. It focuses on the most populated subdistrict (Kuala Belait, Seria, and Liang), where 97% of residents reside.
Participants: A final of 1000 respondents aged 18 years and older were randomly selected from the chosen sub-districts population with a 95% confidence interval and ±3 margin of error. Due to the variable selection, only 673 respondents were available for the analysis.
Outcome measures: SRH was dichotomized with 1 for good health and 0 otherwise. Perception of environmental quality included perceptions of the natural environment (air quality, marine quality, water supply, noise and olfactory pollution) and social

environment (crime). Chi-square and logistic regression models were used to assess the relationship between individuals' perceived environmental quality and SRH.
Results: Most respondents perceived themselves in good SRH (72%). The adjusted logistic regression shows that the perception of air quality (OR=2.20, 95% CI 1.15-4.22, *p*=0.018) and marine resources (OR=1.84, 95% CI 1.24-2.74, =0.002) in their surrounding areas were significantly associated with good SRH. However, other environmental variables were insignificantly associated with SRH. Among control variables, healthy lifestyle and employment had positive associations with good SRH (OR=3.89, 95% CI 1.96-7.71, *p*=0.000 for exercising 3-5 times a week, and OR=1.72, 95% CI 1.09-2.71, *p*=0.021 for being employed). In addition, frequent physical exercise compensated for the negative health impact of environmental pollution.
Conclusions: This study suggests that environmental quality has an important role in SRH. However, a healthy lifestyle measured with the frequency of physical exercise seems to compensate for the adverse environmental effects on SRH.

KEYWORDS: Self-rated health; perceived environmental quality; socio-economic-demographic condition; healthy lifestyle; Brunei Darussalam.

Article Summary

Strengths and limitations of this study

* This study's strength is in providing new empirical evidence on how environmental quality is related to self-rated health, a subjective measure, especially in an oil- and gas-rich Belait district of Brunei Darussalam, one of the Southeast Asian countries.
* Another strength is that it used a representative and a relatively large district sample.
* One limitation is that there is no comparable measure of objective health.
* Perceptions of olfactory and noise pollutants were grouped into one category, and there was only one variable of social environment, therefore, further studies should collect these pollutants and more variables of social environment separately.
* A longitudinal study is important to further examine a causal relationship between perception of environmental quality and health and investigate the dynamics of the relationship.

For peer review only - http://bmjopen.bmj.com/site/about/guidelines.xhtml

INTRODUCTION

Ensuring healthy life is Goal 3 of the 17 sustainable development goals[1]. Health crisis has far-reaching consequences on individuals, families, communities and states. At the individual level, being unhealthy encompasses suffering, deprivation of self-confidence and capability to function, and losing productivity and various opportunities. To some extent, being unhealthy can lead to a premature end of life. What happens to individuals may impact the whole family. Unhealthy or sick family members may lead to a family caregiving crisis and disrupt other family members' social and economic activities. They may even fall into poverty[2]–[4].

Communicable diseases, for example, may even become a pandemic, resulting in a sudden considerable disruption of people's life. At the state level, a health crisis can weaken the heath-care system and destabilize the economy through a contraction in employment, increased state expenditure, and reduced investment. The current COVID-19 pandemic provided evidence of the health crisis' impact on both health and the economy in many countries around the globe[5]–[7].

To be healthy has multi-dimensional factors[8] with complex roles at different levels (individual, families, community and state). Previous studies were mostly concerned with health-related individual and family factors such as medical, biological or hereditary conditions; psychosocial, demographic, social and economic conditions, and healthy lifestyle[9]–[14]. State-level health status factors were only considered when the analyses were concerned with cross-countries differences. For example, level of democratic governance was found to have a positive association with health[15]. Yet, studies within a country or region should also consider the

For peer review only - http://bmjopen.bmj.com/site/about/guidelines.xhtml

health-related community factors, especially the importance of surrounding environmental conditions. This environmental condition can take three forms: built or man-made, natural and social environment[16].

Because it is not easy to measure objective measures of environmental quality at the individual level, this paper utilized its proxy -- perceived environmental quality. Most importantly, objective measures of environmental quality apply to all individuals living in the same environment. Then there is no variation among individuals. Thus, the environment-health relationship does not exist in a small study area. The use of subjective measures of environmental quality can vary from one individual to another. Perception of environmental quality may matter to individuals. Individuals' perception of their environmental quality may say more about a variation in the perceptions of environment and health. This study contributes to new empirical findings. This subjective variable was then used to examine the association between the environmental quality of the surrounding areas and self-rated health status. Similarly, in population-based surveys, health status was assessed subjectively, known as self-rated health (SRH). SRH has been widely used in many countries. SRH is a reliable health indicator used in population-based studies[17], [18] and provides a reliable and acceptable approximation of the overall health outcomes of the population[18]–[20]. It captures the biological, mental, and social states of an individual health[21] and has been shown to be able to explain mortality and morbidity[21], [22], and causes of deaths[14], [23].

Assessing the association between these two subjective domains has been conducted mostly in Western countries[24]–[30], and some Asian countries such as Sri

For peer review only

For peer review only - http://bmjopen.bmj.com/site/about/guidelines.xhtml

Lanka[31], China[16], [32], Japan and South Korea[33]. However, little is known about this relationship among Southeast Asian countries, where countries in this region prospered and developed significantly. Natural environmental issues in this region such as seasonal forest fires, have also been a transboundary concern[34].

Environment is a broad concept and has been measured differently from one study to another. For example, a study in England and Scotland[24] measured neighbourhood environmental conditions related to man-made and social environments such as access to private transportation, physical quality of the residential environment, political climate and political engagement. Similarly, another study in the United Kingdom[28] used these environmental conditions, but the selected variables referred to access to amenities, neighbourhood quality, and neighbourhood disorder. Unlike these studies[24], [28], environmental condition among residents of Chelsea, Massachusetts, in the US was measured differently by asking about the perception of the natural environment regarding air quality, odour and noise, and social environment, including feeling safe, neighbourhood crime, and social cohesion[26]. A study in Sri Lanka[31] examined environmental factors using ventilation problems, water shortage, garbage disposal problems, mosquito threat, stray dogs, and social environment (nuisance from neighbours and overcrowding). A study in China concerning its rapid development examined people's perception of air pollution, water pollution, freshwater resource shortage and green space shortage in relation to self-related health[16].

However, findings from the abovementioned studies were inconclusive. The study in England and Scotland found that most of their selected neighbourhood

For peer review only - http://bmjopen.bmj.com/site/about/guidelines.xhtml

environmental factors did not associate with SRH[24]. Similarly, none of the environmental factors was associated with SRH[31]. In contrast, some studies found that people's poor living environment was likely associated with a higher likelihood of reporting poor SRH[25]–[29], [33]. Urban residents in China who perceived air pollution and noise pollution were more likely to report not in good SRH[32]. Air pollution had health impacts on respiratory diseases in some Southeast Asian countries[35] and non-communicable diseases such as chronic kidney diseases in 194 countries and territories[36]. Different results were also found in China[32] that perceived environmental pollution was not significantly associated with SRH among rural residents of China. Nevertheless, there has been an insufficient number of empirical studies which assessed the impacts of perceived environmental quality on self-rated health among Southeast Asian countries.

With this background, identifying environmental factors on health is crucial to contribute new empirical findings to the debate on the environment-health relationship, more specifically between perceived environmental quality and self-rated health (SRH). A study in China[16] reported that the advantage of examining subjective indicators than objective indicators on environment-health relationships may provide more stories about individuals' life. This study aims to fill in the gap in this relationship by controlling for demographic, socio-economic and lifestyle variables in Brunei Darussalam (hereafter, Brunei). This study does not consider variables measuring built environmental quality[16] due to the unavailability of information. Our primary objectives are, therefore to estimate the prevalence of good SRH and examine the relationship between natural and social environmental qualities

For peer review only - http://bmjopen.bmj.com/site/about/guidelines.xhtml

and SRH accounting for dimensions of socio-economic-demographic and healthy lifestyle variables based on a cross-sectional study. Brunei's development has led to rapid urbanization transforming its green nature and social environment for human needs.

Brunei has also provided its people with high quality of life, as indicated by its high HDI (Human Development Index), which stood at the 43th worldwide in 2018[37]. Its economy is heavily dependent on oil and gas production, with oil and natural gas exports as its major export and its main source of development and wealth since the mid-20th century. Brunei benefits considerably from the world market oil price, making it the second-highest per capita GDP after Singapore among Southeast Asian countries.

METHODS

Design, Setting, Study size and Participants

We used the Strengthening the Reporting of Observational Studies in Epidemiology (STROBE) cross-sectional checklist when writing our report[38]. The paper used an original cross-sectional survey conducted by the Universiti Brunei Darussalam between 17 October and 11 November 2019, before the COVID-19 pandemic spread to this country in March 2020. The survey was conducted in the Belait district, the largest one of the four districts in Brunei in terms of land size. This district was home to around 75,900 persons or 16.5% of 459,500 Brunei's population in 2019[39]. Within this district, three sub-districts were selected, namely Seria, Liang, and Kuala Belait, where the majority of people (97%) reside and the primary sources of the country's

For peer review only - http://bmjopen.bmj.com/site/about/guidelines.xhtml

economy – the production of hydrocarbon resources - are located. They are located in the northern coastal area facing the South China Sea, as seen in Figure 1.

Figure 1 about here

This survey interviewed 1,000 randomly selected respondents aged 18 years and above residing in the chosen subdistrict (or *Mukim*). Based on the 2016 population census, the population aged 18 years and above in Belait was roughly 72 percent data[40]. This sample size was randomly drawn with a 95% confidence level and ±3% margin of error. Face-to-face interviews were performed by 11 trained research assistants. The survey gathered comprehensive information using a pre-tested structured questionnaire covering background characteristics, employment and income, housing, education and aspirations, health, and environmental impact and awareness. The questions were mostly closed-ended, combined with several open-ended ones. Prior to the interviews, respondents were aware and informed about the survey's objectives. The response for each selected variable varied for various reasons such as do not want to answer, do not know, unclear reason and others. For consistency, 673 respondents who completely responded to all questions were selected for this paper. Table 1 below provides further details of the basic characteristics of the sample.

Patient and Public Involvement

Informed consent was provided for which respondents signed a consent form to agree or not to participate voluntarily.

For peer review only - http://bmjopen.bmj.com/site/about/guidelines.xhtml

Research Ethics Approval

The University Research Ethics Committee at Universiti Brunei Darussalam approved its research ethic with the reference number UBD/OAVCR/UREC/Sep19-04.

Variables and Measurements

The Dependent Variable: Self-rated Health (SRH)

Self-rated health (SRH) has been widely used in population-based surveys due to its simplicity as a singular question. Usually, the question took a form such as "In general, would you say that your health is ...?[41]–[43]. The Likert scale was usually used for the response. However, in practice, the question varies slightly in different contexts. For instance, "Would you say your overall physical health is...?" was used by a study in the US [20]. Studies in Japan and Korea used "How would you rate your health?"[17], and in Norway "How is your health at the moment"[44]. In Timor Leste and India, self-rated physical health was measured by more than one variable and therefore expressed as an index[43], [45]. Most of these studies used a Likert scale.

SRH in this paper was assessed with a single question: "Please select the item that accurately describes your overall current health". The response item was a five-point Likert scale (very good, good, fair, poor, very poor). Guided by common practice [16], [20], [24], [26], [46]–[48], this paper classified the responses into a dichotomized dependent variable to measure good SRH by combining very good and good health into 1 and 0 otherwise.

For peer review only - http://bmjopen.bmj.com/site/about/guidelines.xhtml

Focal Variables: Perceived Environmental Quality

Perceptions of environmental quality (*PEQ*), the main independent variables, consisted of natural and social environmental quality. Data on natural environmental quality were collected by asking respondents to state the extent of pollution of air quality, marine resources, other pollutions such as noise, olfactory/odour and others; and social environment was indicated with neighbourhood crime. These variables were measured at different points of the Likert scale. "How would you characterize the air quality in your area?" had three scales (1=very clean, 2=fairly clean, and 3=not clean); the last category was treated as the reference group in the logistic model. Two other questions were asked on the importance of air quality improvement, and commitment to it: "How important is air quality improvement to you?" and "Compared to other people, how would you describe your commitment to preserving and improving air quality?". The improvement in air quality was treated as a dichotomous variable with the value of 1 for very important or somewhat important and 0 for not very important or not important at all. The commitment to improve air quality was treated as a dichotomous variable by giving the value of 1 for much more committed, somewhat more committed, or about the same and 0 for somewhat less committed or much less committed.

Perception on water supply quality and other pollution were Yes-No questions: "Do you have any problem with water supply?" and "Is the dwelling exposed to noise, odour, or pollution problems?". The answer for "yes" was given the value of 1 and 0 otherwise. "How would you describe the condition of the marine resources (e.g., fisheries, coral reefs etc.) in your area today?" This response was treated as a

For peer review only - http://bmjopen.bmj.com/site/about/guidelines.xhtml

dichotomous variable by giving the value of 1 for very good, good, or not good not bad, and 0 for bad or very bad. Only one variable was available for social environmental quality. "Do you consider your area a high, medium or low crime area?" It was treated as a dichotomous variable with 1 for medium or high crime and 0 for low crime.

Control Variables

Demographic and Socio-Economic Variables

Control variables consisted of demographic, socio-economic, and healthy lifestyle variables. Demographic variables included age, sex, marital status, and ethnic group. Sex was treated as a dichotomous with the value of 1 for males and 0 for females. Age was treated as a numerical variable for the logistic model and a categorical variable (18-29, 30-59, and 60-85 years old) for Chi-square analysis. Marital status was categorized into three groups: married, ever married (divorced and widowed) and single/never married, with married as the reference group. Ethnicity consisted of four groups: Brunei Malay, Indigenous Malay, Chinese and Others. As of the Brunei Nationality Act 1961, the Malay group consists of seven ethnic groups: Brunei Malay or Brunei, Belait, Bisaya, Dusun, Kedayan, Murut and Tutong[49]. Brunei Malay is culturally Malay and embraces Islam, similar to the Malay in West Malaysia, while other indigenous Malays are culturally and religiously diverse[50].

Social-economic variables (*SES*) included education, employment status and ownership of the house. Education had four groups (primary, secondary, post-secondary and higher degree). Employment status was categorized into employed,

For peer review only - http://bmjopen.bmj.com/site/about/guidelines.xhtml

unemployed and not in the labour force (i.e., those who are neither in employment nor in unemployment). Being not in the labour force was treated as the reference group. House ownership was a dichotomous variable (1 for owning the house and 0 for not owning the house).

Healthy Lifestyle

Due to limited information, a healthy lifestyle (*HLS*) was measured with a survey question asking about the frequency of doing physical activities: "How often do you play sports or exercise per week?" It had four responses: 1=never, 2= 1 to 2 times, 3=3 to 5 times, and 4= every day. Never playing sport or exercise was the reference category.

Statistical Methods

Chi-square statistics was initially performed through a series of cross-tabulations between self-rated health status and all selected variables. Logistic regression model[51] was used for assessing the association between perceived environmental quality and SRH. Below is the logistics regression model used in this paper.

$$\ln\left(\frac{p}{1-p}\right) = \beta_0 + \beta_j PEQ_j + \beta_k DEM_k + \beta_l SES_l + \beta_m HLS_m + \varepsilon$$

where p refers to probability of good self-rate health (SRH)

PEQ refers to perceived environmental quality with j indicators

DEM refers to demographic control variable, with k indicators

SES refers to socio-economic variables with i indicators

HLS refers to a healthy lifestyle with only 1 indicator

For peer review only - http://bmjopen.bmj.com/site/about/guidelines.xhtml

Following the previous study[17], the model was applied in few stages to examine each group of variables at each stage. This paper has four stages to observe the possible changes played by perceived environmental quality, demographic variables, socio-economic variables and lifestyle variables on SRH. Model 1 assessed the association between SRH and *PEQ* without control variables. Model 2 enriched Model 1 by adding demographic variables, Model 3 further added *SES,* while Model 4 added *HLS* as controls. The *p*=0.05 was selected to determine the significance of the variables. Odds Ratio (OR) and its 95% Confident Intervals (CI) were provided to examine the significance of the relationship.

RESULTS

Sample description

An overview of the characteristics of the respondents based on demographic, socioeconomic variables and environmental quality is presented in Table 1. More than half were female (52.3%) and mature ages between 30-59 years old (51.0%). The majority were married (55.6%), with a significant percentage of single (37.7%). The rest was ever married, either widowed or divorced (6.7%). Brunei Malay accounted for the largest ethnic group (63.3%). More than half attended post-secondary and above (58.5%). About 65% were employed. Less than half did not own the houses they lived in. Regarding a healthy lifestyle, only one fifth never participated in physical exercise, and many of them had their physical activities once or twice per week.

For peer review only - http://bmjopen.bmj.com/site/about/guidelines.xhtml

Table 1. Respondents Characteristics and Comparison in Good SRH

Variable		Frequency	Percent	Good SRH	X^2 *value (p)*
	Total	673	100.0	-	
Self-rated health	Not good	184	27.3	-	
	Good health	489	72.7	-	
Sex	Female	352	52.3	72.4	0.002 (0.964)
	Male	321	47.7	72.9	
Age	18-29	241	35.8	77.2	7.785 (0.020)
	30-59	343	51.0	72.3	
	60-85	89	13.2	61.8	
Marital status	Married	374	55.6	71.9	2.453 (0.293)
	Ever married	45	6.7	64.4	
	Single	254	37.7	75.2	
Ethnic groups	Brunei Malay	426	63.3	74.9	3.292 (0.349)
	Indigenous Malay	116	17.2	70.7	
	Chinese	75	11.1	66.7	
	Others	56	8.3	67.9	
Education	Primary and below	37	5.5	54.1	6.926 (0.074)
	Secondary	242	36.0	74.0	
	Post-secondary & diploma	221	32.8	74.2	
	High degree	173	25.7	72.8	
House ownership	Own house	304	45.2	70.1	1.878 (0.171)
	Not own	369	54.8	74.8	
Employment status	Employed	437	64.9	76.4	12.578 (0.002)
	Unemployed	44	6.5	77.3	
	Not labour force	192	28.5	63.0	
Physical exercise	Never	142	21.1	63.4	22.956 (0.000)
	1-2 times	362	53.8	70.4	
	3-5 times	128	19.0	88.3	
	Everyday	41	6.1	75.6	

Source: Authors' calculation based on data collected for this study.

Perceived Environmental Quality

The analysis of the survey data shows a good perception of air quality in the area the respondents reside. The descriptive statistics show that 80.2% of respondents perceived that they reside in fairly good air quality areas. Only 12.5% of respondents perceived they live in an area with very good air quality. In other words, only a

smaller percentage of respondents (7.3%) perceived they live in air-polluted areas. They expressed the need to improve the air quality, and half of them were committed to it (Table 2). About 77% did not have problems with sources of water supply in their daily life. The majority (73.7%) did not perceive the existence of other pollutions such as noise, olfactory and others (Table 2). However, a lower percentage, more than half of them (53.8%), perceived that the condition of marine resources in the district was not good. Social environmental quality was reported frequently by 71.9% of residents considering their areas have low crime.

Table 2. Differential of Good SRH by Perceived Environmental Quality

		Frequency	Percent	Good SRH	X^2 *value (p)*
Air quality	Very clean	84	12.5	77.4	
	Fairly clean	540	80.2	73.9	12.903 (0.002)
	Not clean	49	7.3	51.0	
Water problem	No problem	517	76.8	74.9	5.410 (0.026)
	Water supply problem	156	23.2	65.4	
Marine	Not good	362	53.8	67.4	10.330 (0.001)
	Good	311	46.2	78.8	
Other pollutions	Not exposed	496	73.7	74.0	1.440 (0.230)
	Exposed to noise, odour, pollution problems	177	26.3	68.9	
Crime	Low crime	484	71.9	73.3	0.296 (0.586)
	Medium & high	189	28.1	70.9	
Air quality improvement	Not very/not important	169	25.1	71.6	0.067 (0.796)
	Important to improve air quality	504	74.9	73.0	
Commitment	Less committed	313	46.5	72.2	0.026 (0.873)
	Committed	360	53.5	73.1	

Source: Authors' calculation based on data collected for this study.

Differential of SRH: Chi-square analysis

Overall, the majority (72.7%) perceived good self-rated health (SRH). The Chi-square analysis presented in Table 2 shows that the prevalence of good SRH significantly

For peer review only - http://bmjopen.bmj.com/site/about/guidelines.xhtml

differed by perceived air quality (X^2=12.9, p=0.002), marine resources (X^2=10.3, p=0.001) and water supply (X^2=5.4, p=0.026). The prevalence of good SRH for respondents living in perceived very clean air areas was higher (77.4%) than those living in fairly clean air areas (73.9%) and much higher than those living in polluted or not clean air areas (52.0%). Perceived air quality was thus positively associated with good SRH. However, the prevalence of good SRH was not significantly different between respondents perceiving very importantly to improve air quality and those perceiving not importantly to do so (Table 2). In other words, commitment to improving air quality was not associated with good SRH, though perceived air quality was positively related to good SRH. This indicates the existence of a knowledge-attitude gap.

The prevalence of good SRH was higher among those living in areas perceived to have good marine resources than those living in areas not having good marine resources (78.8% and 67.4%, respectively, p<0.001). The prevalence of good SRH was different between respondents having exposure to noise, olfactory and other pollutants and those not being exposed to these pollutants (74.0% and 68.9%, respectively), although the Chi-square result shows no significant difference (X^2=1.4, p=0.230).

Prevalence of good SRH was higher among those living in a house without any problems with water supply (74.9%) than those having problems with water supply (65.4%, p<0.005). Social environment condition, measured with the perception of safety from crime, was not associated with good SRH.

For peer review only - http://bmjopen.bmj.com/site/about/guidelines.xhtml

Age mattered for health status. Table 1 shows that the prevalence of good SRH was lower for the older ages than the younger ages. Men and women perceived almost equally good SRH (72.9% and 72.4%, respectively, X^2=0.002, p=0.964). There was also no ethnic differential in the prevalence of good SRH (X^2=3.3, p=0.349).

Among socio-economic variables, employment status is the only variable which significantly differentiated good SRH (X^2=12.6, p=0p=0.002). The prevalence of good SRH for either employed (76.4%) or unemployed (77.3%) was higher than for those who were not in the labour force (63.0%). Marital status, education and home ownership were not significantly associated with SRH.

Physical exercise mattered more (X^2=23.0, p=0.000). Prevalence of good SRH was much higher (88.3%) among respondents exercising regularly between 3 and 5 times per week than those who never did any physical exercise (63.4%).

Logistic regression of self-rated health

There are four logistic regression results presented in Tables 3 and 4. Model 1 examines the association between perception of environmental quality and SRH without controlling for other sets of variables. This model shows that respondents with the perception of living in fairly clean air areas (OR=2.42, 95% CI 1.30 to 4.49, p=0.005) or very clean air areas (OR=2.40, 95% CI 1.07 to 5.38, p=0.034) were more likely to state in good SRH than those living in the areas with air pollution. This pattern was slightly different from the Chi-square result, with respondents in very clean air areas having the highest prevalence of good SRH. While our further examination (not shown in the table) indicates that the difference between these two

For peer review only - http://bmjopen.bmj.com/site/about/guidelines.xhtml

groups was insignificant, the association between perception of air quality and good SRH remains significant.

Table 3. Odds Ratios for Good SRH and Perceived Environmental Quality controlled for Demographic Variables

Variables	Model 1			Model 2		
	OR	95% CI	Sig.	OR	95% CI	Sig.
**Perceived environmental**						
Air quality (ref=Not clean)			0.020			0.024
Very clean	2.40	1.07 to 5.38	0.034	2.37	1.04 to 5.41	0.040
Fairly clean	2.42	1.30 to 4.49	0.005	2.39	1.28 to 4.49	0.007
Marine Resources (ref=Not good)						
Good Marine Resources	1.69	1.18 to 2.44	0.005	1.78	1.22 to 2.60	0.003
Other pollutions (ref=Not expose)						
Exposed Noise, odour, & others	1.03	0.68 to 1.55	0.907	1.02	0.68 to 1.56	0.908
Water supply (ref=No problem)						
Problem with water supply	0.71	0.47 to 1.08	0.110	0.74	0.49 to 1.13	0.166
Crime (ref=Low crime)						
Medium and High crime	0.98	0.67 to 1.45	0.933	0.90	0.61 to 1.34	0.610
Air Quality Improvement (ref=Not important)						
Important	0.96	0.64 to 1.45	0.857	0.96	0.63 to 1.45	0.830
Commitment to Air Quality Improvement (ref=Not committed)						
Committed	1.08	0.75 to 1.54	0.687	1.02	0.71 to 1.46	0.934
**Demographic variables**						
Age (in years)	-	-	-	0.98	0.96 to 0.99	0.001
Sex (ref=Female)						
Male	-	-	-	1.02	0.71 to 1.46	0.913
Ethnic group (ref=Brunei Malay)						0.588
Indigenous Malay	-	-	-	0.82	0.51 to 1.31	0.397
Chinese	-	-	-	0.86	0.49 to 1.51	0.597
Others	-	-	-	0.68	0.37 to 1.28	0.231
Marital status (ref=Married)						0.409
Ever married	-	-	-	0.86	0.42 to 1.74	0.669
Single	-	-	-	0.72	0.44 to 1.19	0.201
Constant	1.02		0.967	3.70		0.018

Notes: Ref = reference group

Source: Authors' calculation based on data collected for this study.

For peer review only - http://bmjopen.bmj.com/site/about/guidelines.xhtml

Table 4. Odds Ratios for Good SRH and Perceived Environmental Quality controlled for Demographic-Socio-Economic and Healthy Lifestyle Variables

Variables	Model 3			Model 4		
	OR	95% CI	Sig.	OR	95% CI	Sig.
**Perceived environmental**						
Air quality (ref=Not clean)			0.029			0.059
Very clean	2.44	1.06 to 5.63	0.036	2.19	0.93 to 5.12	0.072
Fairly clean	2.35	1.25 to 4.44	0.008	2.20	1.15 to 4.22	0.018
Marine Resources (ref=Not good)						
Good Marine Resources	1.77	1.20 to 2.60	0.004	1.84	1.24 to 2.74	0.002
Other pollutions (ref=Not expose)						
Exposed Noise, odour, & others	1.07	0.70 to 1.63	0.771	0.98	0.63 to 1.51	0.911
Water supply (ref=No problem)						
Problem with water supply	0.72	0.47 to 1.11	0.136	0.71	0.46 to 1.10	0.123
Crime (ref=Low crime)						
Medium and High crime	0.90	0.60 to 1.34	0.595	0.97	0.64 to 1.45	0.866
Air Quality Improvement (ref=Not important)						
Important	0.97	0.64 to 1.47	0.874	0.98	0.64 to 1.50	0.926
Commitment to Air Quality Improvement (ref=Not committed)						
Committed	0.99	0.69 to 1.44	0.975	0.92	0.63 to 1.35	0.681
**Demographic variables**						
Age (in years)	0.98	0.96 to 0.99	0.033	0.98	0.97 to 1.00	0.079
Sex (ref=Female)						
Male	0.98	0.68 to 1.42	0.912	0.86	0.59 to 1.26	0.433
Ethnic group (ref=Brunei Malay)			0.599			0.384
Indigenous Malay	0.78	0.49 to 1.26	0.314	0.78	0.48 to 1.26	0.303
Chinese	0.87	0.49 to 1.54	0.632	0.83	0.46 to 1.48	0.519
Others	0.71	0.37 to 1.34	0.287	0.60	0.31 to 1.16	0.126
Marital status (ref=Married)	-		0.585	-		0.552
Ever married	0.89	0.42 to 1.86	0.747	0.77	0.36 to 1.63	0.487
Single	0.77	0.46 to 1.29	0.320	0.79	0.47 to 1.34	0.384
**Socio-economic variables**						
Education (ref=Primary and below)			0.550			0.435
Secondary	1.35	0.60 to 2.99	0.468	1.27	0.56 to 2.88	0.567
Post-secondary and Diploma	1.04	0.438 to 2.46	0.934	0.93	0.39 to 2.26	0.876
Higher education	0.99	0.411to 2.38	0.981	0.87	0.35 to 2.14	0.755
Employment (ref=Not labour force)			0.091			0.068
Employed	1.63	1.05 to 2.53	0.029	1.72	1.09 to 2.71	0.021
Unemployed	1.53	0.66 to 3.57	0.323	1.63	0.68 to 3.89	0.270
House ownership (ref=Not own)						
Own	1.09	0.73 to 1.63	0.669	1.11	0.60 to 1.36	0.626
**Healthy lifestyle** Physical activities (ref=Never)						0.000
1-2 times	-	-	-	1.06	0.67 to 1.68	0.795
3-5 times	-	-	-	3.89	1.96 to 7.71	0.000
Everyday	-	-	-	1.94	0.83 to 4.50	0.124
Constant	1.76		0.466	1.47		0.633

For peer review only - http://bmjopen.bmj.com/site/about/guidelines.xhtml

Source: Authors' calculation based on data collected for this study.

Respondent's opinion and commitment to improving air quality were not associated with SRH (Table 3). Respondents living in areas with perceived good marine resources were more likely to report good SRH than those living in not good condition marine resources (OR=1.69, 95% CI 1.18 to 2.44, *p*=0.005).

However, good health was not significantly associated with respondents' perception of other environmental qualities (water supply; and pollution of noise, olfactory and others). Model 1 changes the significance level of the association between water supply and good SRH into insignificant from the Chi-square result. This indicates that the presence of perception of air quality and marine had a more salient influence on the association.

Neither was the social environmental condition measured by the perception of crime associated with SRH. These findings on the association between perceived environmental quality on good SRH remain the same after controlling for demographic variables (Model 2).

It is worth briefly discussing the findings on demographic variables. Among the selected demographic variables, age was negatively associated with self-rated health (Model 2). The odds of stating good SRH decreased with age (OR=0.98, 95% CI 0.96 to 0.99, *p*=0.001). This is in contrast to the relationship between gender and good SRH with OR=1 and *p*=0.913. In other words, men and women had the same likelihood of reporting good SRH. There was also no difference in the likelihood of reporting good SRH among marital status and ethnic groups. This deserves further study.

For peer review only - http://bmjopen.bmj.com/site/about/guidelines.xhtml

Adding socio-economic variables, in model 3 (Table 4), all associations between perceived environmental quality and SRH remain the same, with a slight change in the magnitude of the perceived air quality and marine resources. The adjusted odds ratios of reporting good SRH were lower in fairly clean air areas (OR=2.35, 95% CI 1.25 to 4.44, *p*=0.008) and higher in very clean air areas (OR=2.44, 95% CI 1.06 to 5.63, *p*=0.036) than the odds ratios presented in unadjusted model (model 1). In other words, model 3 shows a positive relationship between perceived air quality and good SRH, which was similar to the Chi-square result.

The association between perceived good marine resources quality and SRH became stronger (OR=1.77, 95% CI 1.20 to 2.60, *p*=0.004 in model 3 compared to OR=1.69 in model 1). In other words, controlling for socio-economic variables, the association between perceived environmental quality (especially air quality and marine resources) and SRH became stronger. Socio-economic variables thus strengthened the relationship.

Among socio-economic variables, employment status was the only variable significantly associated with good SRH. Our data shows that a large proportion (28.5%) of the respondents were not in the labour market. If bad health is one of the reasons for not joining the labour market, it is not surprising that employed respondents were more likely to report good SRH than non-labour force (OR=1.63, 95% CI 1.05 to 2.53, *p*=0.029). This likelihood was not different between unemployed and non-labour force. In addition, the likelihood of reporting good SRH was not significant for educational attainment and home ownership.

For peer review only - http://bmjopen.bmj.com/site/about/guidelines.xhtml

Finally, model 4 shows that a healthy lifestyle measured with the frequency of doing physical exercise played the most prominent association with SRH. Doing physical exercise three to five times per week was beneficial for good health (OR=3.89, 95% CI 1.96 to 7.71, *p*=0.000, the highest odd ratio). However, the health impact of doing the physical exercise once or twice, or even everyday weekly, was not significantly different from those never doing any exercise. This may indicate that too much exercise has more detrimental effects than benefits on health. However, there is no further information related to physical exercise, such as the duration of exercise, which deserves further research.

Interestingly, the inclusion of physical exercise into the model made the association between perceived air quality and SRH insignificant (*p*=0.059). In addition, the odds ratio for respondents perceiving fairly clean air became smaller (OR=2.20, Model 4), declining from 2.35 (Model 3). The association between age and SRH also became insignificant. In other words, physical exercise played a mediating role in the association between perceived environmental quality and good SRH. The inclusion of physical activity strengthened the association between employment status and good SRH as the OR for employed in Model 4 (OR=1.72, 95% CI 1.09 to 2.71, *p*=0.021) was higher than in Model 3.

DISCUSSION

Environmental variables

This study contributes to the literature by empirically investigating the relationship between perceived environmental quality and SRH for Brunei, a Southeast Asian

For peer review only - http://bmjopen.bmj.com/site/about/guidelines.xhtml

country. Our findings suggest the importance of addressing natural environmental qualities across different dimensions to gauge their differential relationships with SRH. Specifically, the study found that residents who perceived clean air and good marine resources in their surrounding areas were more likely to report good SRH than residents who perceived polluted air and polluted marine resources. Our findings align with a previous study that marine quality had a positive relationship with health, which was measured as an objective health[52]. Marine and coastal areas have been extensively polluted with various forms of plastics such as water bottles, plastic bags, children's toys, wrappers, medical waste and many others. Plastics affect marine resources through the release of microplastics as harmful pollutant[52]. Polluted marine may pollute the fish and other consumable sea commodities such as seaweed, shellfish and others. This may have further implications for physical health as many people consume the edible marine resources collected and caught in the sea.

It is therefore important to pay attention to the health impact of marine resources pollution as the possibility of both onshore and offshore oil spills occurring in oil production areas such as the Belait district is very high. The health effect of oil spills could last long, and residents' exposure to hydrocarbon had detrimental effects on foetal development and infant mortality, as shown in the case of Nigeria, an oil-producing country[53]. However, land-based activities such as mining, agriculture, forestry, industrial activities, household consumption, river-based transportation, and chemical industries can also be a major source of marine pollution[54].

Furthermore, residents who perceived living in clean air areas were more likely to report good health. Our findings are consistent with earlier studies[32], [33], [55].

For peer review only - http://bmjopen.bmj.com/site/about/guidelines.xhtml

Poor air quality could cause respiratory infection[35], [56]. Polluted air was also associated with cardiovascular disease, lung cancer, and lung function. As part of the Southeast Asian region, Brunei is prone to forest fires, especially during the dry season[57], [58]. Seria, one of the selected sub-district area, was the hotspot area for forest fires[57]. Bushfires have also often become a public health concern in other countries such as Australia[59], Canada[60] and the US[61]. In addition, exposure to transboundary haze from surrounding ASEAN countries was occasionally experienced in Brunei[34].

Both indoor and outdoor air pollution have been a major concern for global public health[56]. Open burning of household rubbish is another source of air pollution. Air pollution, measured objectively by particulate molecule (PM_{10}) exceeding the level for good quality of air in Belait, had the longest recorded days among districts[62]. The lessons from various studies around the Ecuadorian Amazon regions were that crude-oil production activities contributed negative health impacts to communities in the form of various cancers such as rectum, skin, kidney, stomach, leukaemia, soft tissue, and cervix[63]. Therefore, policy-making needs to consider the surrounding environment to improve people's health.

At the same time, our findings also indicate that commitment to improve air quality was not important in good SRH, though perceived air quality was positively related to good SRH. Perhaps, the respondents believed that the government could do better in preserving the air quality.

Noise pollution among urban residents of China was positively associated with poor SRH[26], [32]. However, in the Brunei case, our finding reveals that perceived

For peer review only - http://bmjopen.bmj.com/site/about/guidelines.xhtml

olfactory (odour) and noise pollutant was not significantly related to health, although 26.3% of the selected sample perceived to be exposed to olfactory and noise pollutants.

A previous study found that the impact of noise on health was mostly from road traffic[64]. Noise from road traffic may result in nervousness and disturb sleep quality. Nevertheless, as road traffic is relatively low in Brunei and Belait in particular, compared to many other countries, noise pollutants may not have any impact on health in our study. Moreover, literature review[65] revealed that there was limited empirical evidence on the association between olfactory pollutants and health. As such, our finding may indicate that olfactory and noise pollution might be a nuisance but did not necessarily affect health.

Non-environmental variables

Another important finding is that a healthy lifestyle such as frequent physical exercise can compensate for the health impact of perceived environmental quality. Physical activity mediated the relationship between perceived air quality and SRH. Our findings show that if residents actively and regularly practiced physical exercise, the difference in the prevalence of good SRH became insignificant between residents living in perceived clean air and polluted air. In other words, regular physical activity can mitigate the adverse environmental consequences on health. Our findings reveal that regular exercise was positively associated with good SRH, confirmed by earlier studies[22], [41], [47].

Previous studies showed that the association between age and health was non-conclusive. Age was not significantly associated with SRH[26]. On the other hand, in

For peer review only - http://bmjopen.bmj.com/site/about/guidelines.xhtml

our study, age was negatively associated with good SRH when the model was not controlled with physical activity. This means that as people became older, the prevalence of good health declined, supporting earlier studies[24], [32], [41], [46].

However, our finding further suggests a mediating effect of physical activity on the relationship between age and SRH, which turned this relationship insignificant. In other words, for the same level of physical activity, age did no longer differentiate SRH. The result indicates that the older people who exercised regularly may have an insignificant difference in good health compared to younger people. In other words, a physical activity plays a very important role in SRH for all ages.

Another important variable associated with good SRH was employment. Previous studies indicated that work-health relationships can be simultaneous. The relationship can be a result of bias in which only healthy people are willing to work, and unhealthy ones are leaving their jobs. Poor SRH can thus be associated with being the unemployed or increasing risk of job loss[66]. On the other hand, the relationship between employment and health had mixed results; being employed can be associated with good SRH, bad SRH or no relationship[66]. Being employed in poorly paid jobs or stressful jobs can be associated with poor SRH. However, our study found a positive association -- being employed was more likely to be good SRH (OR=1.72, 95% CI 1.09 to 2.71, p=0.021) than being out of the labour force or unemployed.

Methodological limitations of the study

Our study has few limitations. The first limitation is that there is no comparable measure of objective health available at individual level in the sample. The second

For peer review only - http://bmjopen.bmj.com/site/about/guidelines.xhtml

limitation is that the questionnaire recorded information on "olfactory pollutant" and "noise pollutant" in one group. Yet, these pollutants may have different health effects. The third limitation is that there was only one variable of social environment. Future study requires to separate questions for these two pollutants and collect more information measuring social environmental quality. The fourth limitation is that this study has not examined the dynamics of the relationship. It needs a longitudinal study to address this issue.

Conclusion

Our study concludes that perception of marine resources and air quality mattered to the residents' self-rated health status. Therefore, special attention must be spent to improving air quality and marine resources. Moreover, our findings also show that physical exercise and employment play important roles in health. There is a strong need for policy intervention to mitigate the negative health impact of environmental quality through promoting physical exercise and employment. Further studies should explore more comprehensive measurements of environmental quality, healthy lifestyle and employment variables in assessing health and environmental quality.

Acknowledgements

The authors would like to thank Brunei Shell Petroleum (BSP) Sendirian Berhad for providing the funding to conduct this study. The authors would also like to thank Professor Gour Dasvarma for valuable input and constructive comments. We also thank the field research team who worked hard to complete the data collection.

For peer review only - http://bmjopen.bmj.com/site/about/guidelines.xhtml

Author Contributions. ENA designed the concept, analysed data, chose the method, and wrote the manuscript. C-YH supervised project administration and data collection, wrote, reviewed and edited the final draft. LS coordinated the data collection, reviewed and edited the final draft. AT facilitated funding acquisition, analysed data, reviewed the methods and edited the final draft. All authors contributed to read, edit and approve the final draft of the manuscript.

A Competing interests statement. None declared.

Funding statement. The work was supported by a research grant sponsored by the Brunei Shell Petroleum (BSP) Sendirian Berhad with the reference no.: BSP/SI2018.011/CEA4. The funder had no role in the study design, data collection and analysis, decision to publish, or preparation of the manuscript. The authors independently analysed the anonymous data.

Data statement. No data are available to public due to data agreement.

Patient consent for publication Not required.

Informed consent. Respondents did give informed consent.

Word count: 4699 words

References

[1] Ministry of Finance and Economy, *Voluntary national Review Report of Brunei Darussalam*. Ministry of Finance and Economy, Government of Brunei Darussalam, 2020.

[2] H. Buddelmeyer and L. Cai, "Interrelated Dynamics of Health and Poverty in Australia," *SSRN Electron. J.*, no. 4602, 2021, doi: 10.2139/ssrn.1519238.

[3] P. Clarke and G. Erreygers, "Defining and measuring health poverty," *Soc. Sci. Med.*, vol. 244, no. February 2019, p. 112633, 2020, doi: 10.1016/j.socscimed.2019.112633.

[4] A. Ananta, A. I. A. Moeis, H. T. Widianto, H. Yulianto, and E. N. Arifin,

For peer review only - http://bmjopen.bmj.com/site/about/guidelines.xhtml

"Pension and Active Ageing: Lessons Learned from Civil Servants in Indonesia," *Soc. Sci.*, vol. 10, no. 11, p. 436, 2021, doi: 10.3390/socsci10110436.

[5] H. A. Dang, P. Lanjouw, and E. Vrijburg, "Poverty in India in the face of Covid-19: Diagnosis and prospects*," *Rev. Dev. Econ.*, vol. 25, no. 4, pp. 1816–1837, 2021, doi: 10.1111/rode.12833.

[6] S. Olivia, J. Gibson, and R. Nasrudin, "Indonesia in the Time of Covid-19," *Bull. Indones. Econ. Stud.*, vol. 56, no. 2, pp. 143–174, 2020, doi: 10.1080/00074918.2020.1798581.

[7] M. Whitehead, D. Taylor-Robinson, and B. Barr, "Poverty, health, and covid-19," *BMJ*, vol. 372, pp. 1–2, 2021, doi: 10.1136/bmj.n376.

[8] B. A. Craig *et al.*, "The association between self-rated health and social environments, health behaviors and health outcomes: A structural equation analysis," *BMC Public Health*, vol. 18, no. 1, pp. 1–10, 2018, doi: 10.1186/s12889-018-5323-y.

[9] N. Hoeymans, E. J. M. Feskens, D. Kromhout, and G. A. M. Van Den Bos, "The contribution of chronic conditions and disabilities to poor self- rated health in elderly men," *Journals Gerontol. - Ser. A Biol. Sci. Med. Sci.*, vol. 54, no. 10, pp. 501–506, 1999, doi: 10.1093/gerona/54.10.M501.

[10] H. W. Lee, M. Song, J. J. Yang, and D. Kang, "Determinants of poor self-rated health in Korean adults with diabetes," *J. Prev. Med. Public Heal.*, vol. 48, no. 6, pp. 287–300, 2015, doi: 10.3961/jpmph.15.048.

[11] Y. Li *et al.*, "Determinants of self-rated health among an older Tibetan population in a Chinese plateau area: analysis based on the conceptual

For peer review only

framework for determinants of health," *BMC Public Health*, vol. 21, no. 1, pp. 1–11, 2021, doi: 10.1186/s12889-021-10359-x.

[12] J. H. Kim, K. R. Kim, K. H. Cho, K. B. Yoo, J. A. Kwon, and E. C. Park, "The association between sleep duration and self-rated health in the Korean general population," *J. Clin. Sleep Med.*, vol. 9, no. 10, pp. 1057–1064, 2013, doi: 10.5664/jcsm.3082.

[13] H. Pikhart, *Social and Psychosocial Determinants of Self-Rated Health in Central and Eastern Europe*. 2002.

[14] M. Wuorela, S. Lavonius, M. Salminen, T. Vahlberg, M. Viitanen, and L. Viikari, "Self-rated health and objective health status as predictors of all-cause mortality among older people: A prospective study with a 5-, 10-, and 27-year follow-up," *BMC Geriatr.*, vol. 20, no. 1, pp. 1–7, 2020, doi: 10.1186/s12877-020-01516-9.

[15] P. M. Krueger, K. Dovel, and J. T. Denney, "Democracy and self-rated health across 67 countries: A multilevel analysis," *Soc. Sci. Med.*, vol. 143, pp. 137–144, 2015, doi: 10.1016/j.socscimed.2015.08.047.

[16] H. Chen, Y. Liu, Z. Zhu, and Z. Li, "Does where you live matter to your health? Investigating factors that influence the self-rated health of urban and rural Chinese residents: Evidence drawn from Chinese General Social Survey data," *Health Qual. Life Outcomes*, vol. 15, no. 1, pp. 1–11, 2017, doi: 10.1186/s12955-017-0658-0.

[17] J. H. Park and K. S. Lee, "Self-rated health and its determinants in Japan and South Korea," *Public Health*, vol. 127, no. 9, pp. 834–843, 2013, doi:

For peer review only

For peer review only - http://bmjopen.bmj.com/site/about/guidelines.xhtml

10.1016/j.puhe.2012.12.012.

[18] V. Baidin, C. J. Gerry, and M. Kaneva, “How Self-Rated is Self-Rated Health? Exploring the Role of Individual and Institutional Factors in Reporting Heterogeneity in Russia,” *Soc. Indic. Res.*, vol. 155, no. 2, 2021, doi: 10.1007/s11205-020-02604-4.

[19] S. Cullati, S. Mukhopadhyay, S. Sieber, A. Chakraborty, and C. Burton-Jeangros, “Is the single self-rated health item reliable in India? A construct validity study,” *BMJ Glob. Heal.*, vol. 3, no. 6, pp. 1–12, 2018, doi: 10.1136/bmjgh-2018-000856.

[20] C. K. Haddock *et al.*, “The validity of self-rated health as a measure of health status among young military personnel: Evidence from a cross-sectional survey,” *Health Qual. Life Outcomes*, vol. 4, pp. 1–9, 2006, doi: 10.1186/1477-7525-4-57.

[21] E. L. Idler and Y. Benyamini, “Self-Rated Health and Mortality : A Review of Twenty-Seven Community Studies Author (s): Ellen L . Idler and Yael Benyamini Source : Journal of Health and Social Behavior , Vol . 38 , No . 1 (Mar ., 1997), pp . 21-37 Published by : American Sociologic,” *J. Health Soc. Behav.*, vol. 38, no. 1, pp. 21–37, 1997.

[22] H. Jradi, Z. Alharbi, and Y. Mohammad, “Self-rated health among Saudi women: Association with morbidity, lifestyle, and psychosocial factors,” *J. Epidemiol. Glob. Health*, vol. 8, no. 3–4, pp. 183–188, 2018, doi: 10.2991/j.jegh.2018.04.100.

[23] T. L. Vie, K. O. Hufthammer, E. Meland, and H. J. Breidablik, “Self-rated

For peer review only

health (SRH) in young people and causes of death and mortality in young adulthood. A prospective registry-based Norwegian HUNT-study," *SSM - Popul. Heal.*, vol. 7, no. January, p. 100364, 2019, doi: 10.1016/j.ssmph.2019.100364.

[24] S. Cummins, M. Stafford, S. Macintyre, M. Marmot, and A. Ellaway, "Neighbourhood environment and its association with self rated health: Evidence from Scotland and England," *J. Epidemiol. Community Health*, vol. 59, no. 3, pp. 207–213, 2005, doi: 10.1136/jech.2003.016147.

[25] L. Hale *et al.*, "Perceived neighborhood quality, sleep quality, and health status: Evidence from the Survey of the Health of Wisconsin," *Soc. Sci. Med.*, vol. 79, no. 1, pp. 16–22, 2013, doi: 10.1016/j.socscimed.2012.07.021.

[26] J. Y. Ou, J. L. Peters, J. I. Levy, R. Bongiovanni, A. Rossini, and M. K. Scammell, "Self-rated health and its association with perceived environmental hazards, the social environment, and cultural stressors in an environmental justice population," *BMC Public Health*, vol. 18, no. 1, pp. 1–10, 2018, doi: 10.1186/s12889-018-5797-7.

[27] K. V. Patel, K. Eschbach, L. L. Rudkin, M. K. Peek, and K. S. Markides, "Neighborhood context and self-rated health in older Mexican Americans," *Ann. Epidemiol.*, vol. 13, no. 9, pp. 620–628, 2003, doi: 10.1016/S1047-2797(03)00060-7.

[28] W. Poortinga, F. D. Dunstan, and D. L. Fone, "Perceptions of the neighbourhood environment and self rated health: A multilevel analysis of the Caerphilly Health and Social Needs Study," *BMC Public Health*, vol. 7, pp. 1–

For peer review only

For peer review only - http://bmjopen.bmj.com/site/about/guidelines.xhtml

10, 2007, doi: 10.1186/1471-2458-7-285.

[29] S. Roh, Y. Jang, D. A. Chiriboga, K. H. Kwag, S. Cho, and K. Bernstein, "Perceived neighborhood environment affecting physical and mental health: A study with Korean American older adults in New York City," *J. Immigr. Minor. Heal.*, vol. 13, no. 6, pp. 1005–1012, 2011, doi: 10.1007/s10903-011-9492-3.

[30] U. Saikia, M. M. Dodd, J. Chalmers, G. Dasvarma, and S. Schech, "COVID-19, individual wellbeing and multidimensional poverty in the state of South Australia," *PLoS One*, vol. 16, no. 6 June, pp. 1–17, 2021, doi: 10.1371/journal.pone.0252898.

[31] B. Perera, T. Østbye, and C. Jayawardana, "Neighborhood environment and self-rated health among adults in southern Sri Lanka," *Int. J. Environ. Res. Public Health*, vol. 6, no. 8, pp. 2102–2112, 2009, doi: 10.3390/ijerph6082102.

[32] T. Yang, "Association between perceived environmental pollution and health among urban and rural residents-a Chinese national study," *BMC Public Health*, vol. 20, no. 1, pp. 1–10, 2020, doi: 10.1186/s12889-020-8204-0.

[33] J. Liu, Y. Luo, W. Haller, B. Vander Mey, and E. Granberg, "Neighborhood environments and self-rated health in Mainland China, Japan and South Korea," *PLoS One*, vol. 13, no. 9, pp. 1–17, 2018, doi: 10.1371/journal.pone.0204910.

[34] S. Q. Dotse, L. Dagar, M. I. Petra, and L. C. De Silva, "Influence of Southeast Asian Haze episodes on high PM10 concentrations across Brunei Darussalam," *Environ. Pollut.*, vol. 219, pp. 337–352, 2016, doi: 10.1016/j.envpol.2016.10.059.

[35] L. M. T. Luong, P. D. Sly, P. K. Thai, and D. Phung, "Impact of ambient air

For peer review only - http://bmjopen.bmj.com/site/about/guidelines.xhtml

pollution and wheeze-associated disorders in children in Southeast Asia: A systematic review and meta-analysis," *Rev. Environ. Health*, vol. 34, no. 2, pp. 125–139, 2019, doi: 10.1515/reveh-2018-0079.

[36] B. Bowe, Y. Xie, T. Li, Y. Yan, H. Xian, and Z. Al-Aly, "Estimates of the 2016 global burden of kidney disease attributable to ambient fine particulate matter air pollution," *BMJ Open*, vol. 9, no. 5, 2019, doi: 10.1136/bmjopen-2018-022450.

[37] United Nations, *Human Development Report 2019*. 2019.

[38] E. von Elm, D. G. Altman, M. Egger, S. J. Pocock, P. C. Gøtzsche, and J. P. Vandenbroucke, "The strengthening the reporting of observational studies in epidemiology (STROBE) statement: Guidelines for reporting observational studies," *Int. J. Surg.*, vol. 12, no. 12, pp. 1495–1499, 2014, doi: 10.1016/j.ijsu.2014.07.013.

[39] B. D. Department of Economic Planning and Statistics, "Report of the Mid-Year Population Estimates 2020," 2021.

[40] DEPD, *Population and Housing Census Update Final Report 2016*. Department of Economic Planning and Development (DEPD), Ministry of Finance and Economy, 2018.

[41] A. Rütten *et al.*, "Self reported physical activity, public health, and perceived environment: Results from a comparative European study," *J. Epidemiol. Community Health*, vol. 55, no. 2, pp. 139–146, 2001, doi: 10.1136/jech.55.2.139.

[42] A. K. Harschel, L. A. Schaap, S. Iwarsson, V. Horstmann, and S. Tomsone, "Self-rated health among very old people in European countries: An

For peer review only - http://bmjopen.bmj.com/site/about/guidelines.xhtml

explorative study in Latvia and Sweden," *Gerontol. Geriatr. Med.*, no. January-December, 2015, doi: 10.1177/2333721415598432.

[43] N. A. Government of Assam, UNDP, "Assam Human Development Report 2014: Managing Diversities, Achiveing Human Development," *Gov. Assam*, pp. 1–306, 2014.

[44] T. L. Vie, K. O. Hufthammer, T. L. Holmen, E. Meland, and H. J. Breidablik, "Is self-rated health in adolescence a predictor of prescribed medication in adulthood? Findings from the Nord Trøndelag Health Study and the Norwegian Prescription Database," *SSM - Popul. Heal.*, vol. 4, no. November 2017, pp. 144–152, 2018, doi: 10.1016/j.ssmph.2017.11.010.

[45] UNDP, *National Human Development Report 2018 - Timor-Leste*. 2018.

[46] J. W. Choi, J. Kim, E. Han, and T. H. Kim, "Association of employment status and rated health among income with self- - waged workers with disabilities in South Korea : population- - based panel study," pp. 1–6, 2019, doi: 10.1136/bmjopen-2019-032174.

[47] W. J. Stronegger, S. Titze, and P. Oja, "Perceived characteristics of the neighborhood and its association with physical activity behavior and self-rated health," *Heal. Place*, vol. 16, no. 4, pp. 736–743, 2010, doi: 10.1016/j.healthplace.2010.03.005.

[48] M. Wen, L. C. Hawkley, and J. T. Cacioppo, "Objective and perceived neighborhood environment, individual SES and psychosocial factors, and self-rated health: An analysis of older adults in Cook County, Illinois," *Soc. Sci. Med.*, vol. 63, no. 10, pp. 2575–2590, 2006, doi: 10.1016/j.socscimed.2006.06.025.

For peer review only - http://bmjopen.bmj.com/site/about/guidelines.xhtml

[49] Department of Economic Planning and Statistics, *Brunei darussalam statistical yearbook 2020*. 2021.

[50] S. P. Loo, "Ethnicity and Educational Policies in Malaysia and Brunei Darussalam," vol. 6, no. 2, pp. 180–192, 2009, [Online]. Available: https://www.academia.edu/710806/Ethnicity_and_educational_policies_in_Malaysia_and_Brunei_Darussalam.

[51] D. W. Hosmer, S. Lemeshow, and R. X. Sturdivant, *Applied Logistic Regression: Third Edition*. 2013.

[52] R. Krishna, M. Mohan, P. Mallika, and D. Costa, "Microplastics in ballast water as an emerging source and vector for harmful chemicals , antibiotics , metals , bacterial pathogens and HAB species : A potential risk to the marine environment and human health," *Mar. Pollut. Bull.*, vol. 149, no. July, p. 110525, 2019, doi: 10.1016/j.marpolbul.2019.110525.

[53] A. Bruederle and R. Hodler, "Effect of oil spills on infant mortality in Nigeria," *Proc. Natl. Acad. Sci. U. S. A.*, vol. 116, no. 12, pp. 5467–5471, 2019, doi: 10.1073/pnas.1818303116.

[54] K. A. Willis *et al.*, "Cleaner seas: reducing marine pollution," *Rev. Fish Biol. Fish.*, vol. 8, 2021, doi: 10.1007/s11160-021-09674-8.

[55] P. Liu, J. Wang, X. Wang, W. Nie, and F. Zhen, "Measuring the association of self-perceived physical and social neighborhood environment with health of Chinese rural residents," *Int. J. Environ. Res. Public Health*, vol. 18, no. 16, pp. 1–12, 2021, doi: 10.3390/ijerph18168380.

[56] N. Khaltaev and S. Axelrod, "Chronic respiratory diseases global mortality

For peer review only - http://bmjopen.bmj.com/site/about/guidelines.xhtml

trends, treatment guidelines, life style modifications, and air pollution: Preliminary analysis," *J. Thorac. Dis.*, vol. 11, no. 6, pp. 2643–2655, 2019, doi: 10.21037/jtd.2019.06.08.

[57] M. Radojevic and H. Hassan, "Air quality in Brunei Darussalam during the 1998 haze episode," *Atmos. Environ.*, vol. 33, no. 22, pp. 3651–3658, 1999, doi: 10.1016/S1352-2310(99)00118-1.

[58] S. Shahriar, Z. El-Said M. M., S. Safwanah N. M., H. Kho J., N. N. D. H. M. F. Lee, and N. H. H. Hasim, "Risk assessment for forest fire in Brunei Darussalam," *MATEC Web Conf.*, vol. 258, p. 05033, 2019, doi: 10.1051/matecconf/201925805033.

[59] N. Borchers Arriagada, A. J. Palmer, D. M. J. S. Bowman, G. G. Morgan, B. B. Jalaludin, and F. H. Johnston, "Unprecedented smoke-related health burden associated with the 2019–20 bushfires in eastern Australia," *Med. J. Aust.*, vol. 213, no. 6, pp. 282–283, 2020, doi: 10.5694/mja2.50545.

[60] C. J. Matz *et al.*, "Health impact analysis of PM2.5 from wildfire smoke in Canada (2013–2015, 2017–2018)," *Sci. Total Environ.*, vol. 725, p. 138506, 2020, doi: 10.1016/j.scitotenv.2020.138506.

[61] N. Fann *et al.*, "The health impacts and economic value of wildland fire episodes in the U.S.: 2008–2012," *Sci. Total Environ.*, vol. 610–611, pp. 802–809, 2018, doi: 10.1016/j.scitotenv.2017.08.024.

[62] E. N. Aziz, A. Kasem, W. S. Haji Suhaili, and P. Zhao, "Convolution recurrent neural network for daily forecast of PM10 concentrations in Brunei Darussalam," *Chem. Eng. Trans.*, vol. 83, no. February, pp. 355–360, 2021, doi:

For peer review only - http://bmjopen.bmj.com/site/about/guidelines.xhtml

10.3303/CET2183060.

[63] G. Coronel Vargas, W. W. Au, and A. Izzotti, "Public health issues from crude-oil production in the Ecuadorian Amazon territories," *Sci. Total Environ.*, vol. 719, p. 134647, 2020, doi: 10.1016/j.scitotenv.2019.134647.

[64] S. Geravandi *et al.*, "Noise Pollution and Health Effects," *Jundishapur J. Heal. Sci.*, vol. 7, no. 1, pp. 1–15, 2015, doi: 10.5812/jjhs.25357.

[65] V. Guadalupe-Fernandez *et al.*, "Industrial odour pollution and human health: a systematic review and meta-analysis," *Environ. Heal. A Glob. Access Sci. Source*, vol. 20, no. 1, pp. 1–21, 2021, doi: 10.1186/s12940-021-00774-3.

[66] L. Antonisse and R. Garfield, "The Relationship Between Work and Health: Findings from a Literature Review," *Issue Br.*, no. August, 2018, [Online]. Available: https://www.kff.org/medicaid/issue-brief/the-relationship-between-work-and-health-findings-from-a-literature-review/.

Figure 1. Geographical location of the studied areas

For peer review only

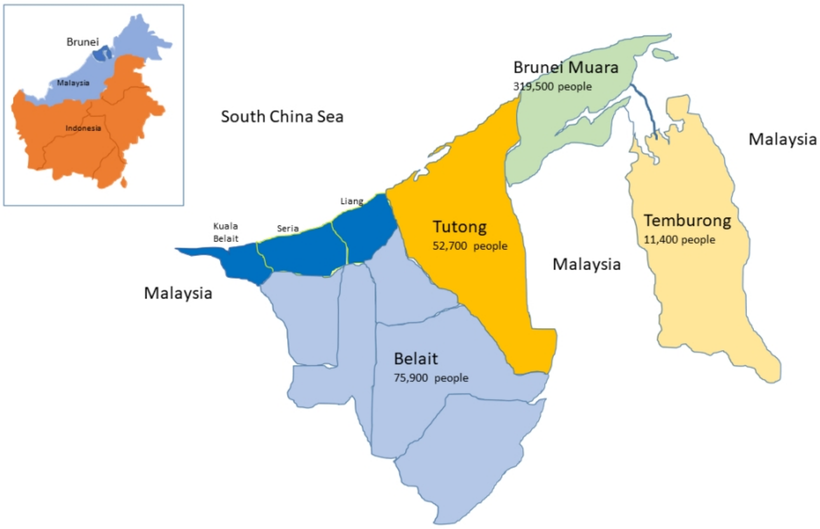

Figure 1. Geographical location of the studied areas

338x190mm (96 x 96 DPI)

Reporting checklist for cross sectional study.

Based on the STROBE cross sectional guidelines.

Instructions to authors

Complete this checklist by entering the page numbers from your manuscript where readers will find each of the items listed below.

Your article may not currently address all the items on the checklist. Please modify your text to include the missing information. If you are certain that an item does not apply, please write "n/a" and provide a short explanation.

Upload your completed checklist as an extra file when you submit to a journal.

In your methods section, say that you used the STROBE cross sectionalreporting guidelines, and cite them as:

von Elm E, Altman DG, Egger M, Pocock SJ, Gotzsche PC, Vandenbroucke JP. The Strengthening the Reporting of Observational Studies in Epidemiology (STROBE) Statement: guidelines for reporting observational studies.

		Reporting Item	Page Number
**Title and abstract**			
Title	#1a	Indicate the study’s design with a commonly used term in the title or the abstract	1

For peer review only

Abstract	#1b	Provide in the abstract an informative and balanced summary of what was done and what was found	1-2
**Introduction**			
Background / rationale	#2	Explain the scientific background and rationale for the investigation being reported	3-6
Objectives	#3	State specific objectives, including any prespecified hypotheses	6
**Methods**			
Study design	#4	Present key elements of study design early in the paper	7
Setting	#5	Describe the setting, locations, and relevant dates, including periods of recruitment, exposure, follow-up, and data collection	7
Eligibility criteria	#6a	Give the eligibility criteria, and the sources and methods of selection of participants.	8
	#7	Clearly define all outcomes, exposures, predictors, potential confounders, and effect modifiers. Give diagnostic criteria, if applicable	8-11
Data sources / measurement	#8	For each variable of interest give sources of data and details of methods of assessment (measurement). Describe comparability of assessment methods if there is more than one group. Give information separately for for exposed and unexposed groups if applicable.	8-11

For peer review only

Bias	#9	Describe any efforts to address potential sources of bias	7-8
Study size	#10	Explain how the study size was arrived at	8
Quantitative variables	#11	Explain how quantitative variables were handled in the analyses. If applicable, describe which groupings were chosen, and why	10-11
Statistical methods	#12a	Describe all statistical methods, including those used to control for confounding	10-13
Statistical methods	#12b	Describe any methods used to examine subgroups and interactions	10-13
Statistical methods	#12c	Explain how missing data were addressed	8
Statistical methods	#12d	If applicable, describe analytical methods taking account of sampling strategy	-
Statistical methods	#12e	Describe any sensitivity analyses	-
**Results**			
Participants	#13a	Report numbers of individuals at each stage of study—eg numbers potentially eligible, examined for eligibility, confirmed eligible, included in the study, completing follow-up, and analysed. Give information separately for for exposed and unexposed groups if applicable.	13
Participants	#13b	Give reasons for non-participation at each stage	13

For peer review only

Participants	#13c	Consider use of a flow diagram	
Descriptive data	#14a	Give characteristics of study participants (eg demographic, clinical, social) and information on exposures and potential confounders. Give information separately for exposed and unexposed groups if applicable.	13
Descriptive data	#14b	Indicate number of participants with missing data for each variable of interest	13
Outcome data	#15	Report numbers of outcome events or summary measures. Give information separately for exposed and unexposed groups if applicable.	13
Main results	#16a	Give unadjusted estimates and, if applicable, confounder-adjusted estimates and their precision (eg, 95% confidence interval). Make clear which confounders were adjusted for and why they were included	13-23
Main results	#16b	Report category boundaries when continuous variables were categorized	13-23
Main results	#16c	If relevant, consider translating estimates of relative risk into absolute risk for a meaningful time period	13-23
Other analyses	#17	Report other analyses done—e.g., analyses of subgroups and interactions, and sensitivity analyses	

Discussion

Key results	#18	Summarise key results with reference to study objectives	23-24

For peer review only

Limitations	#19	Discuss limitations of the study, taking into account sources of potential bias or imprecision. Discuss both direction and magnitude of any potential bias.	13-23
Interpretation	#20	Give a cautious overall interpretation considering objectives, limitations, multiplicity of analyses, results from similar studies, and other relevant evidence.	13-23
Generalisability	#21	Discuss the generalisability (external validity) of the study results	23
**Other Information**			
Funding	#22	Give the source of funding and the role of the funders for the present study and, if applicable, for the original study on which the present article is based	24

None The STROBE checklist is distributed under the terms of the Creative Commons Attribution License CC-BY. This checklist can be completed online using https://www.goodreports.org/, a tool made by the EQUATOR Network in collaboration with Penelope.ai

For peer review only
